# PmiRExAt: plant miRNA expression atlas database and web applications

**DOI:** 10.1093/database/baw060

**Published:** 2016-04-13

**Authors:** Anoop Kishor Singh Gurjar, Abhijeet Singh Panwar, Rajinder Gupta, Shrikant S. Mantri

**Affiliations:** ^1^Computational Biology Laboratory, National Agri Food Biotechnology Institute (NABI), Mohali, Punjab, India; ^2^HPC-Tech, Center for Development of Advance Computing (C-DAC), Pune, Maharashtra, India

## Abstract

High-throughput small RNA (sRNA) sequencing technology enables an entirely new perspective for plant microRNA (miRNA) research and has immense potential to unravel regulatory networks. Novel insights gained through data mining in publically available rich resource of sRNA data will help in designing biotechnology-based approaches for crop improvement to enhance plant yield and nutritional value. Bioinformatics resources enabling meta-analysis of miRNA expression across multiple plant species are still evolving. Here, we report PmiRExAt, a new online database resource that caters plant miRNA expression atlas. The web-based repository comprises of miRNA expression profile and query tool for 1859 wheat, 2330 rice and 283 maize miRNA. The database interface offers open and easy access to miRNA expression profile and helps in identifying tissue preferential, differential and constitutively expressing miRNAs. A feature enabling expression study of conserved miRNA across multiple species is also implemented. Custom expression analysis feature enables expression analysis of novel miRNA in total 117 datasets. New sRNA dataset can also be uploaded for analysing miRNA expression profiles for 73 plant species. PmiRExAt application program interface, a simple object access protocol web service allows other programmers to remotely invoke the methods written for doing programmatic search operations on PmiRExAt database.

**Database URL:** http://pmirexat.nabi.res.in.

## Introduction

Discovery of functional endogenous microRNAs (miRNAs), which negatively regulate gene expression at the post-transcriptional level in eukaryotes, has dramatically increased in the recent past. Software, tools and web servers enabling large scale RNA-seq and small RNA (sRNA)-seq expression meta-analysis along with comparative and integrative interpretation have started to proliferate. RNASeqExpressionBrowser (1) and RNA-Seq Atlas (2) offers gene expression analysis and visualization, MIRPIPE (3) supports quantification of miRNAs in niche model organisms lacking genomic sequences, mirEX^2^ (4) supports pri-miRNA expression analysis for *Arabidopsis thaliana, Hordeum* *vulgare and Pellia endiviifolia*, omiRas (5) is used for differential expression (DE) between two given conditions by uploading sRNA sequencing data and PsRobot (6) takes sRNA sequence fasta or plain text files as input. omiRas and PsRobot require genome sequences for analysis and prediction of new miRNA. miRNA play important role in plant development during different growth stages and stress conditions. Understanding gene regulatory networks involving plant miRNA is critical to design biotechnology-based approaches for crop improvement (7). Here, we report PmiRExAt, a new online database resource that provides the most comprehensive comparative view yet of plant miRNAs (miRs) expression in multiple tissues and development stages of wheat (W), rice (R) and maize (M).

## Data sources, data mining and analysis

### Non-redundant miRNA collection

**Figure 1.**
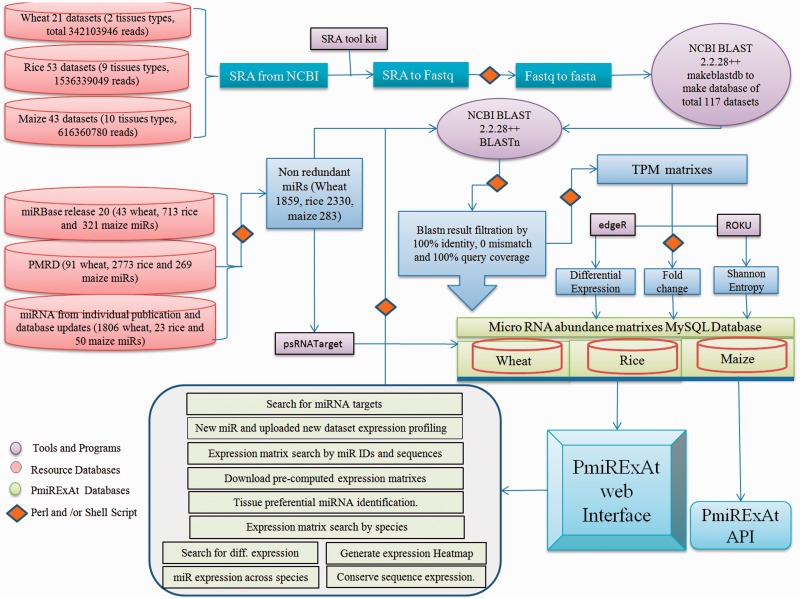
Methodology flow chart showing source of raw data collected, scripts and programmes used in processing files to generate results, database of pre-computed values of processed files.

In this study, the miRNA sequences of three majorly cultivated food crops namely wheat, rice and maize were retrieved from miRBase (release 20) (8), plant miRNA database (PMRD) (9) and recent publications. miRNA sequence redundancy was removed using perl script. One thousand eight hundred and fifty-nine non-redundant (NR) out of 2045 redundant miRNA of wheat, 2330 NR out of 3509 redundant miRNA of rice and 283 NR out of 630 redundant miRNA of maize were analysed further (Figure 1, Table 1 and Supplementary Table S1).

**Table 1.** Dataset and miR statistics^a^

Plant species	Wheat	Rice	Maize
PMRD	91	2773	269
miRBase(release 20)	43	713	321
NR known miRNA from PMRD and miRBase(release 20)	93	2309	233
miR families	75	2066	38
Recent publication miRNA added	1806 (10–14, miRBase (release 21), PNRD)	23 (15)	50 (16–19)
Total NR miRNA (miRBase + PMRD + publication miRs)	1859	2330	283
Datasets analysed	21	53	43
Total number of reads analysed	342 103 946	1 536 339 049	616 360 780

a This table briefs about the miRNA source and their number of families, NR miRNAs, number of datasets for a species and total number of reads in these available datasets.

### sRNA sequencing libraries (datasets) collection

The next generation sequencing (NGS) data of sRNA of wheat, rice and maize were collected from sequence read archive (SRA) database publicly available from National Center of Biotechnology Information (NCBI) website (http://www.ncbi.nlm.nih.gov/sra) (20). Twenty-one sRNA datasets (10, 21–24) for four types of wheat tissues (leaf, spike, generic sample and whole plant), 53 sRNA datasets (25–32) for nine types of rice tissues (whole plant, root, shoot, leaf, panicle, anther, embryo, endosperm and seedling) and 43 sRNA datasets (19, 33–40) for 11 types of maize tissues (whole plant, root, shoot, leaf, ear, anther, tassel, pollen, silk, 5-day-old coleoptile and seedling), comprising in total 2.4+ billion sequence reads were collected (Figure 1, Table 1 and Supplementary Table S1). SRA files were converted into fasta format using fastq_dump function of fastx-toolkit and fastq to fasta using perl script and finally these fasta files were converted into databases by makeblastdb command of NCBI basic local search alignment tool (BLAST) 2.2.28+ package. Respective species NR miRNA were BLASTn against NGS reads databases and results were filtered on the basis of perfect match for developing expression count matrixes (Figure 1). The size distribution of miRNA was estimated by adding expression count of same size miRNAs and taking percent value against total count of expressing miRNA in particular library (Supplementary Figure S1).

### Development of miRNA expression matrix

SRA datasets were converted into BLAST databases by multiple steps of file processing using SRA toolkit and NCBI BLAST 2.2.28+ package (41). Collected NR mature miRNA were used as query against respective wheat, rice and maize sRNA databases. BLASTn program and in-house shell scripts were used for computing miRNA abundance, following stringent criteria of 100% identity, 0 mismatch and 100% miRNA sequence coverage with sRNA database reads.

### Data normalization and visualization

Normalization was done by converting hit counts into transcript per million (TPM = number of miR count in dataset × 1 000 000/total reads in dataset). Heatmaps were developed after log_2_ transformation of TPM values. Normalized expression data (TPM) was sorted on ordinal basis and distributed in 10 categories according to the respective species miRNA numbers for each library/dataset. The heatmaps were developed for each species showing category 1–10 (Supplementary Figure S2 and Table S2).

### Identification and profiling of conserved miRNA

Conserved miRNA between W, R and M were identified on the basis of 100% sequence similarity. We found 51 conserved sequences in WRM (Supplementary Table S1). These 51 miRNA were profiled against all 117 datasets of WRM (wheat-rice-maize). Forty-three miRNA of wheat out of 51 conserved miRNA were showing cumulative abundance above 100, likewise 48 of rice and 44 of maize (Supplementary Figure S3). Conserved miRNA sequences belong to 24 miRNA families (miR156, miR159, miR160, miR164, miR166, miR167, miR168, miR169, miR171, miR172, miR2118, miR319, miR390, miR393, miR394, miR395, miR396, miR399, miR408, miR437, miR444, miR528, miR827 and miRf10461). Composition and length distribution of conserved miRNAs between W, R and M were plotted (Supplementary Figure S4). We also identified 1639 unique miR in wheat, 2182 unique miR in rice and 137 unique miR in maize. Targets for these miRNA were predicted using psRNAtarget (42) tool at default parameters (Supplementary Table S3). Targets were predicted against available Unigene data files on psRNATarget interface (Supplementary Table S3).

Conserved miRNA correlation matrix was also calculated for all datasets (Supplementary Table S4).

### Differential expression analysis

EdgeR package (43) was used to calculate DE of miRNA. Library-wise DE analysis was performed using normalized TPM values of each library.

### Tissue preferential analysis

**Figure 2.**
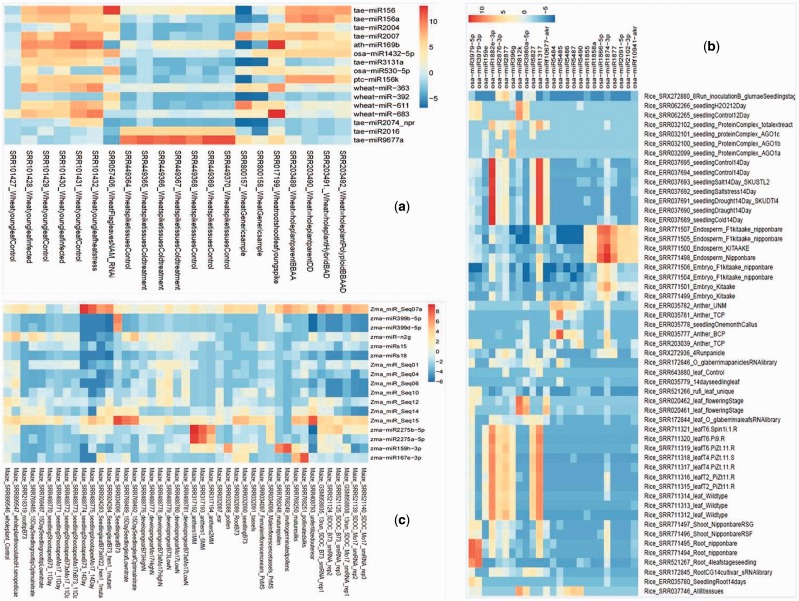
Tissue preferential expression heatmap of wheat (**A**), rice (**B**) and maize (**C**). The tissue preferential miRNA were filtered on the basis of fold change (above 80-fold) expression as compared with other miRNA expression and the fold change values were calculated by cumulative TPM values of one tissue versus average of cumulative TPM of other tissues.

miRNA showing tissue preferential expression were screened by the cumulative TPM 80-fold greater than the mean TPM from other tissues (44) along with Shannon entropy calculations using ROKU package (45). We used default parameter of ROKU viz. upperlimit was kept at default value of 0.25 (specifying the maximum percent of tissue as outlier to each miRNA). ROKU picks tissue-specific patterns from expression data of different tissues and it ranks genes by its overall tissue-specificity using Shannon entropy and an outlier detection method for detecting tissues specific to each gene. Shannon entropy was introduced by Claude Shannon for use in communications technology. It is a measure of the information content. Using the combined approach, we found 14 miRNA preferentially expressing in leaves and 2 in spikes of wheat, whereas in rice 2 miRNA in root, 10 in leaf, 5 in anther and 8 in endosperm and in maize 1 miRNA in shoot, 2 in leaf, 2 in anther, 10 in ear, 1 in pollen and 1 in silk (Figure 2, Table 2). EdgeR package was also used for computing tissue-wise DE for pair of tissue of interest verses mean TPM from other tissues. Logarithmic fold change (logFC), logarithmic counts per million (logCPM), *P*-value and false discovery rate (FDR) values for such cases are available in Supplementary Table S5.

**Table 2.** Tissue preferential miRNA list on the basis of fold change^a^

Tissue	Wheat tissue preferential miR			Rice tissue preferential miR			Maize tissue preferential miR		
	miR_ID	Fold change	Shannon entropy	miR_ID	Fold change	Shannon entropy	miR_ID	Fold change	Shannon entropy
Root	NA			osa-miR3979-5p	322.5	0.21	NA		
				osa-miR3979-3p	655.3	0.18			
Shoot	NA			NA			Zma_miR_Seq07a	109.71	0.45
Leaf	tae-miR156	105.65	0.07	osa-miR159e	129.142	0.76	zma-miR399b-5p	185.88	0.35
	tae-miR156a	134.46	0.06	osa-miR1882e-3p	415.469	0.54	zma-miR399d-5p	94.79	0.6
	tae-miR2004	339.46	0.02	osa-miR2876-3p	176.546	0.77			
	tae-miR2007	1023.48	0.01	osa-miR2877	131.767	0.79			
	ath-miR169b	134.88	0.06	osa-miR396g	146.339	0.92			
	osa-miR1432-5p	134.13	0.06	osa-miR812k	5539.62	0.82			
	tae-miR3131a	235.69	0.03	osa-miR3980a-5p	136.328	0.8			
	osa-miR530-5p	176.08	0.05	osa-miR5827	149.19	0.6			
	ptc-miR156k	196.82	0.04	osa-miR1317	118.808	0.78			
	wheat-miR-363	99.74	0.08	osa-miRf10677-akr	139.762	1.06			
	wheat-miR-392	81.89	0.09						
	wheat-miR-611	447.63	0.02						
	wheat-miR-683	198.24	0.04						
	tae-miR2074_npr	91.96	0.08						
Spike	tae-miR2016	108.98	0.07	NA			NA		
	tae-miR9677a	4098.54	0.003						
Panicle	NA			NA			NA		
Anther	NA			osa-miR5484	255.586	0.27	zma-miR2275b-5p	104.59	0.47
				osa-miR5485	796.998	0.07	zma-miR2275a-5p	271.5	0.25
				osa-miR5486	167.182	0.43			
				osa-miR5487	219.912	0.35			
				osa-miR5490	107.212	0.65			
Embryo	NA			NA			NA		
Endosperm	NA			osa-miR1855	87.5798	0.66	NA		
				osa-miR1858a	128.149	0.49			
				osa-miR1866-5p	364.02	0.12			
				osa-miR1874-3p	187.517	0.23			
				osa-miR1877	299.926	0.17			
				osa-miR2091-5p	273.874	0.25			
				osa-miR2102-3p	226.394	0.28			
				osa-miRf10941-akr	105.026	0.54			
Ear	NA			NA			zma-miR-n2g	84.26	0.55
							zma-miRs15	85.98	0.52
							zma-miRs18	95.91	0.53
							Zma_miR_Seq01	145.48	0.37
							Zma_miR_Seq04	89.07	0.5132029
							Zma_miR_Seq06	238.282	0.2583411
							Zma_miR_Seq10	93.1634	0.558418
							Zma_miR_Seq12	220.186	0.2781626
							Zma_miR_Seq14	383.77	0.213312
							Zma_miR_Seq15	233.921	0.2566291
Tassel	NA			NA			NA		
Pollen	NA			NA			zma-miR159h-3p	93.3895	0.5971372
Silk	NA			NA			zma-miR167e-3p	117.691	0.4833388
Coleoptile	NA			NA			NA		

a NA,  not available.

### New reported miRNA profiling

To capture latest research insights, miRNA expression matrix were computed for novel plant miRNA from recent publications viz. miRNA sequences of wheat (10–14, 46–48), rice (15) and maize (19, 16–18) (Supplementary Table S5), which are not submitted in any public database like miRBase (release 20) and PMRD. Meanwhile, miRBase (version 21) was also released in June 2014, so we also collected miRNA from it. Total 1806 W, 23 R, 50 M novel miRNA were collected. We compared these miRNA sequences with the miRBase (release 20) and PMRD miRNA and made a NR sequences file for each species. These NR new miRNA were profiled against 21 W, 53 R and 43 M datasets. For each of these miRNA targets were predicted by psRNATarget (Supplementary Table S3).

### PmiRExAt database architecture and web interface

PmiRExAt is created with a motive to make miRNA expression database searching easier and user friendly. The web portal is designed with responsive web design approach aimed at crafting it to provide an optimal viewing experience. PmiRExAt has been developed using open source Web 2.0 technologies to enhance the user experience at the web portal. It is developed using Java EE 6 standard and with model–view–controller (MVC) software pattern. MySQL database is used at backend. PmiRExAt uses power of Ajax to asynchronously call server and to provide results on the same page without page refreshing. We have made use of Hibernate object-relational mapping which consistently offers superior performance over straight Java Database Connectivity (JDBC) code in terms of runtime performance and is designed to work in an application server cluster and deliver a highly scalable architecture.

**Figure 3.**
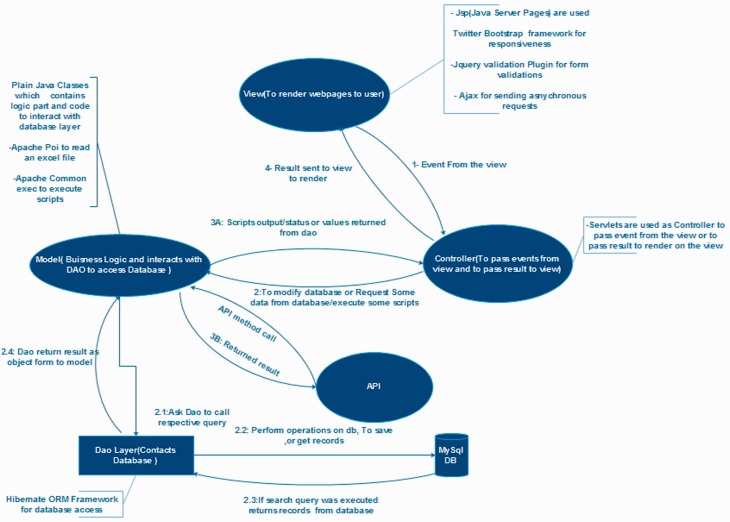
Architectural diagram of PmiRExAt**.** This architectural diagram displays basic design concept of PmiRExAt in MVC architectural pattern and collaboration of the MVC components.

PmiRExAt uses Highcharts application program interface (API) (http://www.highcharts.com) to generate heatmap (Figure 3) and it also use Morpheus API (http://www.broadinstitute.org/cancer/software/morpheus/) for clustering. PmiRExAt uses cocktail of different web technologies and other third party libraries to provide the user a pleasant experience at PmiRExAt. It uses Bootstrap front end framework to support various screen sizes and Ajax to update web pages asynchronously by exchanging small amounts of data with the server behind the scenes. This means that only parts of a web page get updated without reloading the whole page and thus eliminates the need for unnecessary page reloads.

PmiRExAt uses jQuery Tag-it plugin to handle multi-tag fields as well as tag suggestions/auto-complete which thus relieves the user of remembering all miRNAs and dataset names; auto completer automatically suggests all names present at PmiRExAt database as soon as user starts typing for names. For generating the heatmaps, it uses Highcharts charting library which generates interactive and dynamic maps. On hovering the cursor over the heatmap, it displays related information on Tooltip for each point on the heatmap.

PmiRExAt also has an API which has published functionalities provided by the web interface to other software components, which want to use already present functionalities of the web interface. API offers application-components like getting all sequences, species or to perform search on PmiRExAt database using multiple search criteria.

**Figure 4.**
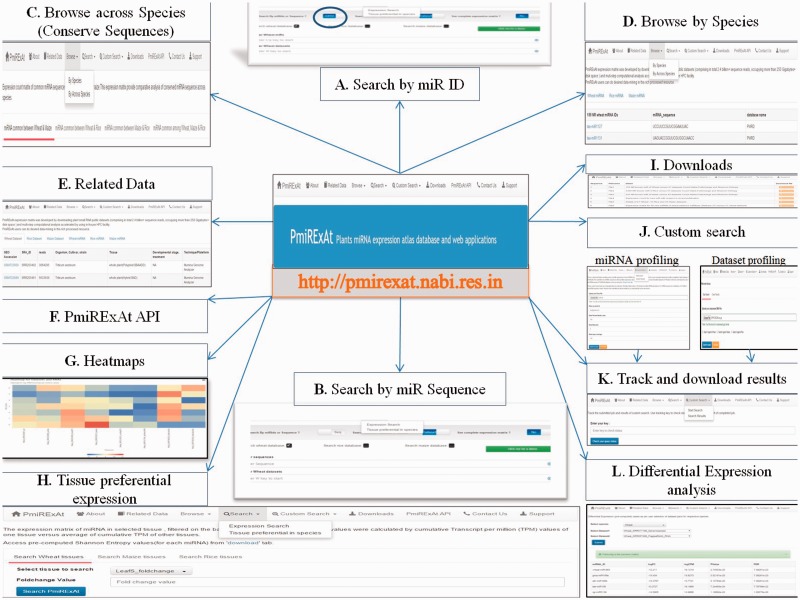
PmiRExAt features (web screen shots). (**A**) Search by miR ID: meant to search a miR expression by providing the miRNA Id. (**B**) Search by miRNA sequence: meant to search a miR expression by providing the miRNA sequence as available in miRBase (release 20, 21), PMRD, PNRD or as given in added publications. (**C**) Browse expression of conserved miRNA: sequences across wheat, rice and maize. (**D**) Browse expression of individual miRNA: in all datasets of particular species. (**E**) Related data: information of datasets and the miRNA sequences can be explored hereby using hyperlinks of miRNA and datasets. (**F**) PmiRExAt API: to use PmiRExAt API, user need to create a web service client in respective platform/language in which user want to use PmiRExAt API. (**G**) Heatmap: expression visualization is supported by heatmaps which is based on log TPM values. (**H**) Tissue preferential expression: the expression matrix of miRNA in selected tissue, filtered on the basis of fold change. (**I**) Download section: the pre-computed matrixes of all data and information of datasets can be downloaded here. (**J**) Custom search: this is a advanced feature enabling users to profile expression of novel miRNA against available 117 datasets or profile 73 plant species miRNA in user uploaded new dataset. (**K**) Job tracking: as user submit the job, system generates a key which is useful in tracking the job status and downloading the results. (**L**) DE analysis: search DE of miRNA by choosing dataset pair or browse DE between pair of control verses condition libraries.

There are multiple tabs on web interface which provides the desired browsing, download and custom search options (Figure 4). PmiRExAt users can do desired data mining in this rich processed resource. Apart from availability of intuitive web server interface, PmiRExAt also caters a simple object access protocol (SOAP) web service which allows other programmers to remotely invoke the methods written for doing search operations on database. Quick start guide (Supplementary File S1) will help users in using web interface and API.

### Comparative analysis of web resources for miRNA expression analysis

sRNA-seq data can be analysed in many ways to find out different aspects of research. Many tools have been developed to analyse data for miRNA expression. Here, we compared features of such available web resources against PmiRExAt to highlight the advantages offered by PmiRExAt. See feature comparison in Table 3.

**Table 3.** Comparative feature between PmiRExAt and other web resources of miRNA expression analysis

S.No.	Feature	mirEX/miREX^2^ (4, 49)	PsRobot (6)	omiRas (5)	MIRPIPE (3)	PmiRExAt
1	Browse pre-computed expression matrixes	No	No	No	No	Yes
2	Species genome required for analysis	No	Yes	Yes	Yes	No
3	Sequence conservation analysis	Yes	Yes	No	No	Yes
4	API	No	No	No	No	Yes
5	Publically available datasets expression count database	No	No	No	No	Yes
6	Direct downloads of expression matrixes and heatmaps	Yes	No	No	No	Yes
7	Filter tissue preferential expressing miR on the basis of Shannon entropy and fold change	No	No	No	No	Yes
8	Contains novel miR reported in latest publication expression data	No	No	No	No	Yes

## Usage and utility

### Search miRNA expression by miRNA IDs or sequences

A NR database of wheat 1859 miRNA, rice 2330 miRNA and maize 283 miRNA was developed from miRBase (release 20, 21), PMRD, plant non-coding RNA database (PNRD) and few miRNA from the publications (Table 1). On the basis of  > 1000 TPM in individual datasets, 45 miRNA in wheat, 55 miRNA in rice and 27 miRNA of maize were considered highly expressing miRs. There were many miRNA which were showing zero cumulative abundance (576/1859 wheat, 320/2330 rice and 23/283 maize).

### Search miRNA expression in particular tissue of species

Expression of miRNA in a particular tissue can be searched by choosing the tissues of analysed datasets of selected species. User can select one or more than one tissue at a time. After clicking on search button, the expression count matrix will be displayed on interface. User can save expression matrix by ‘Export table data’ button and user can also click on the hyperlink of miRs which will lead to the source database miRBase (release 21) and PMRD of miRNA for getting more information about the miRNA precursor, stem loop structure, function and its target. User can also generate expression heatmap by clicking on ‘Generate heatmap’ button and can also generate clustered heatmap. User can download the heatmap in different picture formats like jpeg, pdf, etc. from ‘chart context menu’ at upper most right-hand side of heatmap.

### Custom search for newly detected miRNA sequences in 117 datasets

Maximum five novel sequences can be uploaded at a time for computing their expression matrix against 117 WRM datasets. For this user will have to select ‘Start Search’ from drop down menu of ‘Custom search’ then user can input miRNA sequences in fasta format. User has to enter a functional email ID for receiving the result. User can also customize BLASTn parameters viz. percent identity, mismatch and query coverage or user can choose default values. After user has submitted the job, a random key unique to each job is generated on interface that can be used to track the running job or downloading the results.

### Custom search of miRNAs expression in new library of sRNA sequences

Newly generated sRNA libraries can be analysed for the PmiRExAt miRs and all other plant species miRNA sequences of miRBase (release 21). Here, user needs to register to get benefits of this facility. After registering the user needs to login and upload the SRA file in zip format and choose the desired plant species to develop expression matrix. Custom search feature is also facilitated with tracking the running job status or download the results by entering the key generated at the time of job submission.

### SOAP API and client

There is link to access SOAP web service and Wsdl for API. SOAP message can be formulated and parsed in any chosen languages by application developers. This functionality will be helpful to other programmers/software components to connect to PmiRExAt API.

### Download files

All the processed data contained in database that is used to generate the expression tables and heatmaps can be downloaded.

## Conclusion and future work

PmiRExAt database interface has the following unique features: (i) to search miRNA expression by miRNA ID/s or sequence/s. (ii) To search miRNA expression in particular datasets or tissue/s. (iii) To filter tissue preferentially expressing miRNA. (iv) ‘Browse’ or ‘Search’, ‘DE’ on the basis of edgeR calculation. (v) To browse miRNA expression across species. (vi) To compute and profile expression of newly detected miRNA sequence in 117 datasets, and also new datasets can be uploaded for expression profiling with NR miRNAs of WRM and other 70 plant species mature miR from miRBase (release 21, June 2014). (vii) High-resolution heatmaps are generated on the web interface that helps in visualization and interpretations. This web resource and service will help plant science community in studying expression patterns of miRNAs. This website and web service is free and open to all users. Meta-analysis of the publicly available sRNA-seq datasets showed significant expression patterns of several miRs. Data mining in this developed resource has already led to identification of tissue preferential expressing and conserved miRNA. PmiRExAt will help in exploring public sRNA-seq expression data to find supporting evidence for users’ findings and hypotheses. These expression profiles can be used as a proxy for relative expression levels of miRNA sequences. It will aid in studying plant miRNA gene function by studying where, when and in response to what these miRNA are expressed.

As we expect this project to get bigger in near future, so PmiRExAt is developed keeping an eye on the scalable aspects of the datasets viz. species, miRs, etc. We will keep adding novel miRNA sequences and new sRNA libraries of wheat, rice and maize for better comparative analysis. We will also be adding other agri-food plant species in PmiRExAt database. We will further classify datasets on the basis of developmental stage for more specificity in comparative analysis of miRNA. Micro RNA expression matrices will be useful for studying miRNA regulatory networks in plants.

## Supplementary data

Supplementary data are available at *Database* Online.

Supplementary Data
