# Supplementary material for: PmiRExAt: plant miRNA expression atlas database and web applications
Source: Database (Oxford). 2016 Apr 13;2016:baw060. doi: 10.1093/database/baw060 (PMC4830907; doi:10.1093/database/baw060)
Supplement: Supplementary Data [file supp_2016_baw060_index.html]

Supplementary Data 

# PmiRExAt: plant miRNA expression atlas database and web applications

## Supplementary Data

files

- Supplementary Data - zip file
