# Supplementary material for: PmiRExAt: plant miRNA expression atlas database and web applications
Source: Database (Oxford). 2016 Apr 13;2016:baw060. doi: 10.1093/database/baw060 (PMC4830907; doi:10.1093/database/baw060)
Supplement: Supplementary Data [file supp_baw060_suppl_data.zip › Supplementary doc file 1.docx]

| National agri - food biotechnology institute (NABI), Mohali, INDIA. |
| --- |
| **Quick start guide for PmiRExAt: Plant miRNA Expression Atlas Database** |
| 1)Web Interface 2) SOAP API and Client |
|  |
| User manual V1.0 |
|  |

December, 2015

| Address: C- 127, Industrial Area, Phase VIII, Sector 71, SAS Nagar, Mohali, Punjab-160071. |
| --- |

**About Document**

This document will serve to teach users about how to get started with PmiRExAt quickly. It will serve the purpose of a detailed manual and objectives. This guide is meant for users who want to understand components involved in PmiRExAt. It is only meant for users to initiate specific tasks in PmiRExAt or learn usage of specific searches of PmiRExAt. It can be used by a person who wants to use PmiRExAt irrespective of previous computational or bioinformatics application exposure.

In case of any doubt or unresolved issue that is not covered in this document please send an email to **ict@nabi.res.in.**

**INDEX**

| **S. No** | **Topic** | **Page** |
| --- | --- | --- |
|  | | |
| **A)** | About PmiRExAt | 4 |
|  |  |  |
| **B)** | Web Interface use cases | 5 |
|  | Use Case 1: miRNA expression search by miRNA IDs and datasets | 5 |
|  | Use Case 2: miRNA expression search by miRNA sequences and datasets | 7 |
|  | Use Case 3: Search miRNA or sequence expression in particular tissue of species | 8 |
|  | Use Case 4: Study complete miRNA expression for individual species | 9 |
|  | Use Case 5: Filter tissue preferentially expressing miRNA on the basis of fold-change | 10 |
|  | Use Case 6: Filter tissue preferentially expressing miRNA on the basis of Shannon Entropy | 11 |
|  | Use Case 7: Get differentially expressing miRNAs | 12 |
|  | Use Case 8: Browse species wise detail of miRNA analysed and their targets in PmiRExAt | 13 |
|  | Use Case 9: Browse across species miRNA expression matrix | 15 |
|  | Use Case 10: Browse latest publication miRNA expression | 16 |
|  | Use Case 11: Browse tissue preferential miRNA expression | 17 |
|  | Use Case 12: Browse miRNA differential expression | 18 |
|  | Use Case 13: Custom search for newly detected miRNA sequences in 117 datasets | 19 |
|  | Use Case 14: Custom search miRNA profiling for newly generated sRNA library | 20 |
|  | Track the running job status and download the results | 21 |
|  | Description about tabs | 22 |
|  |  |  |
| **C)** | SOAP API and Client | 23 |
|  | Overview | 23 |
|  | **Getting started** | 23 |
|  | Methods Provided by PmiRExAt API | 23 |
|  | Sample Code | 26 |
|  |  |  |
| **D)** | Contact us | 28 |
|  |  |  |
| **E)** | References | 29 |

1. **About PmiRExAt**

The PmiRExAt is designed to be used by plant science community working on small RNA. The input miRNA can be searched in database and to fetch the expression count matrix with an option to generate heatmap. To understand how to start using the interface, user can use example set at interface by clicking on it to automatically show example inputs.

**Salient features**

- Search miRNA expression by miRNA IDs or sequences.
- miRNA expression in particular tissue.
- miRNA expression for individual species.
- Filter tissue preferentially miRNA.
- MicroRNA differential expression by edgeR.
- Browse species wise miRNA expression.
- Browse across species miRNA expression.
- Custom search for newly detected miRNA sequences in 117 datasets**.**

1. **Web Interface use Cases**

This tool can be used by following ways:

**Use Case-1: miRNA expression search by miRNA IDs and datasets, Figure1**

**Step1**-

1. Click 'Search' button, the drop down menu shows option to select either I) expression search or II) tissue preferential expression in species (1a).
2. Select 'Expression search' from drop down menu. Here user needs to select species viz. wheat (1b), rice (1c), maize (1d) or Arabidopsis (1e) for miR id (1f).
3. User can type the first few letters (1g) of miRNA ID and the available menu of miRNA IDs appear as a drop down. The user can select the miRs from them e.g. 't' for wheat miRNAs, 'o' for rice miRNAs and 'z' for maize miRNAs.
4. The user can provide a comma separated list of miRNA IDs in a text file as input to interface (1h). List of all miRNA present in PmiRExAt database can be accessed on 'Related data' tab.

**Step2-**

1. Select datasets. As user toggle for datasets (2a), user can select desired datasets in the box (2b) below miRNA ID box or can give a list of comma separated datasets in a text file (2c).
2. A drop down menu of all datasets opens up as the user types the first letter of plant species name e.g. W for wheat, R for rice and M for maize. List of all datasets present in PmiRExAt database can be accessed on 'Downloads' tab file "Details of 21 wheat, 53 rice and 43 maize datasets".

**Step3-** Click on search button (3), the expression count matrix will be displayed on interface.

**Step4-** User can save expression matrix by 'Export table data' button (4) and user can also click on the hyperlink of miRs which will lead to the source database miRBase and PMRD of miRNA for more information about the miRNA precursor, stem loop structure, function and its target.

**Step5-** User can generate expression heatmap by clicking on 'Generate heatmap' button (5) and option for clustered heatmap (6).

**Step6-** User can download the heatmap in different picture formats like jpeg, pdf etc from "chart context menu' at upper most right hand side of heatmap.

Note: In the heatmap colored boxes, user can see the miRNA ID, dataset name as well as TPM value as user moves the mouse arrow (tooltip).


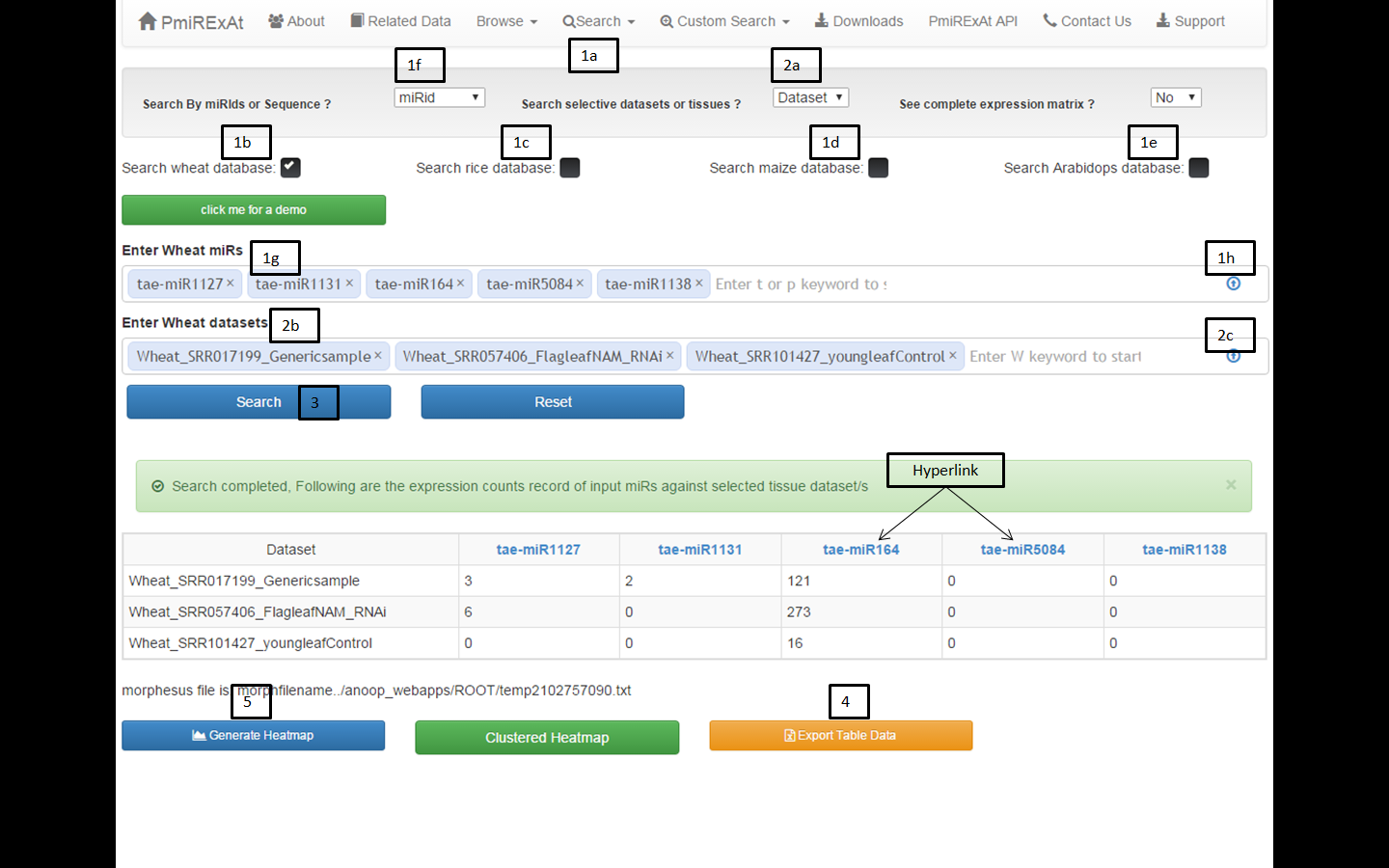


6

Figure 1: Screenshot showing steps to be followed for use case-1.

**Use Case-2: miRNA expression search by miRNA sequences and datasets, Figure2**

**Step1-**

1. On clicking the Search button, the drop down shows options to select either I) 'Expression Search' or II) 'Tissue preferential expression in species' (1a).
2. Select 'Expression Search' from drop down menu. Here the user needs to select species viz. wheat (1b), rice (1c), maize (1d) or Arabidopsis (1e) for miRNA sequence (1f).
3. User can put sequence (1g) of known miRNA and to input another sequence just press space, now it will be ready to paste next miRNA in the box or user can upload a fasta file on interface (1h). List of all miRNA with their sequence present in PmiRExAt database, can be accessed on 'Related data' tab. (Figure2)

Now follow Step 2 to 6 as mentioned in case1.


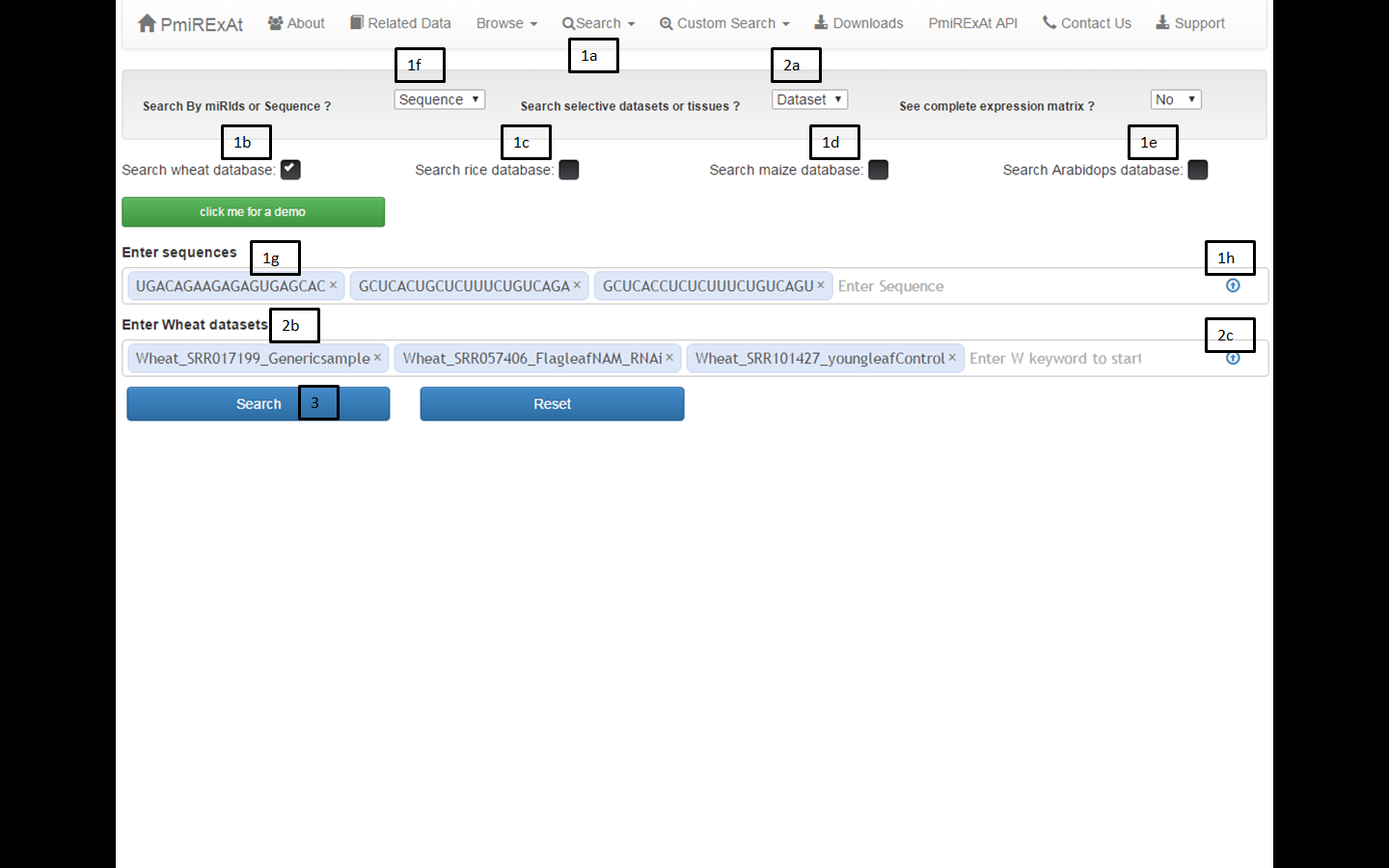


Figure 2: Screenshot showing steps to be followed for use case-2.

**Use Case3: Search miRNA or sequence expression in particular tissue of species, Figure3**

**Step1-** Choose miRNA id or sequence as mentioned in case1 and case2 (step1).

**Step2-** Toggle the dataset to tissue button (2a).

**Step3-** Selects the tissues of analyzed datasets of selected species according to his/her choice (3a-d). User can select one or more than one tissue at a time.

Now follow Step 3 to 6 as mentioned in case1.


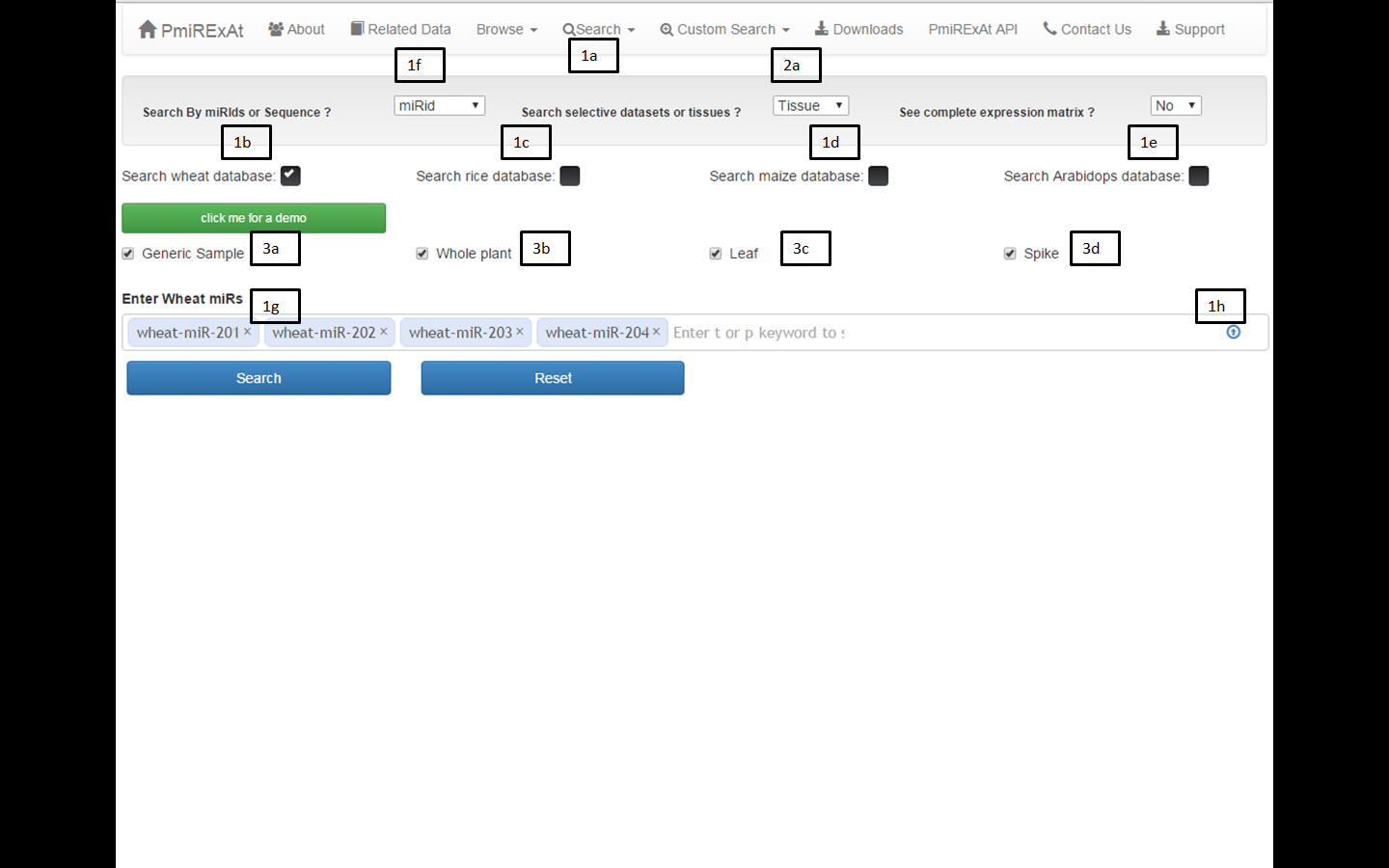


Figure 3: Screenshot showing steps to be followed for use case-3.

**Use Case-4: Study complete miRNA expression for individual species, Figure4**

**Step1-**

1. On clicking Search button, the drop down shows options to select either I) 'Expression Search' or II) 'Tissue preferential expression in species' or III) ‘Differential Expression’ 1(a). Select 'Expression Search' drop down menu.
2. Select ‘Yes’ 'at ‘See complete expression matrix' (1b)

**Step2-** Chooses any one from four buttons that appear on screen (2a), (2b), (2c) or (2d) to see whole expression matrix of the respective species miRNA.

Now follow Step 4 to 6 as mentioned in case1.


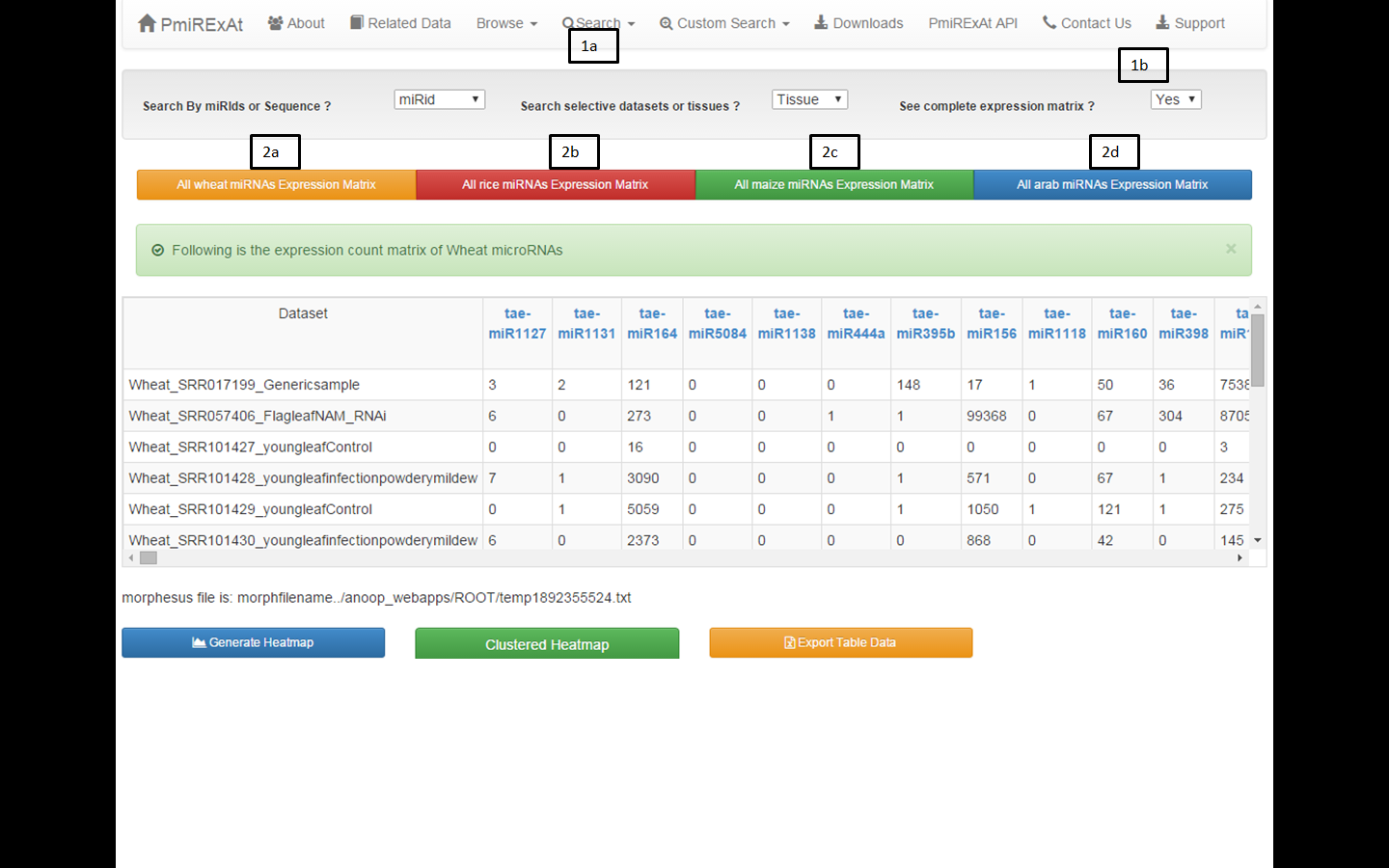


Figure 4: Screenshot showing steps to be followed for use case-4.

**Use Case-5: Filter tissue preferentially expressing miRNA on the basis of fold-change, Figure5**

**Step1-**

1. On clicking the Search button, the drop down shows options to select either I) 'Expression Search' or II) 'Tissue preferential expression in species' (1a) or III) ‘Differential Expression’. Select 'Tissue preferential expression in species' drop down menu.
2. Select your search on the basis of Fold-change (1b)

**Step2-** Click 'Search wheat tissues' (2a) or 'Search rice tissues' (2b) or 'Search maize tissues' (2c) or Search Arabidopsis tissues (2d).

**Step3-**

1. Choose tissue of interest (3a) for available expression data.
2. Input the fold change threshold value for filtering the miRNA expression by giving a numerical value in the text box (3b). The fold change was calculated by dividing the Cumulative TPM values of each tissue datasets by average of other tissues cumulative TPM values (Guo, Z. et al. 2014).

Now click the search button and follow Step 4 to 6 as mentioned in case1.


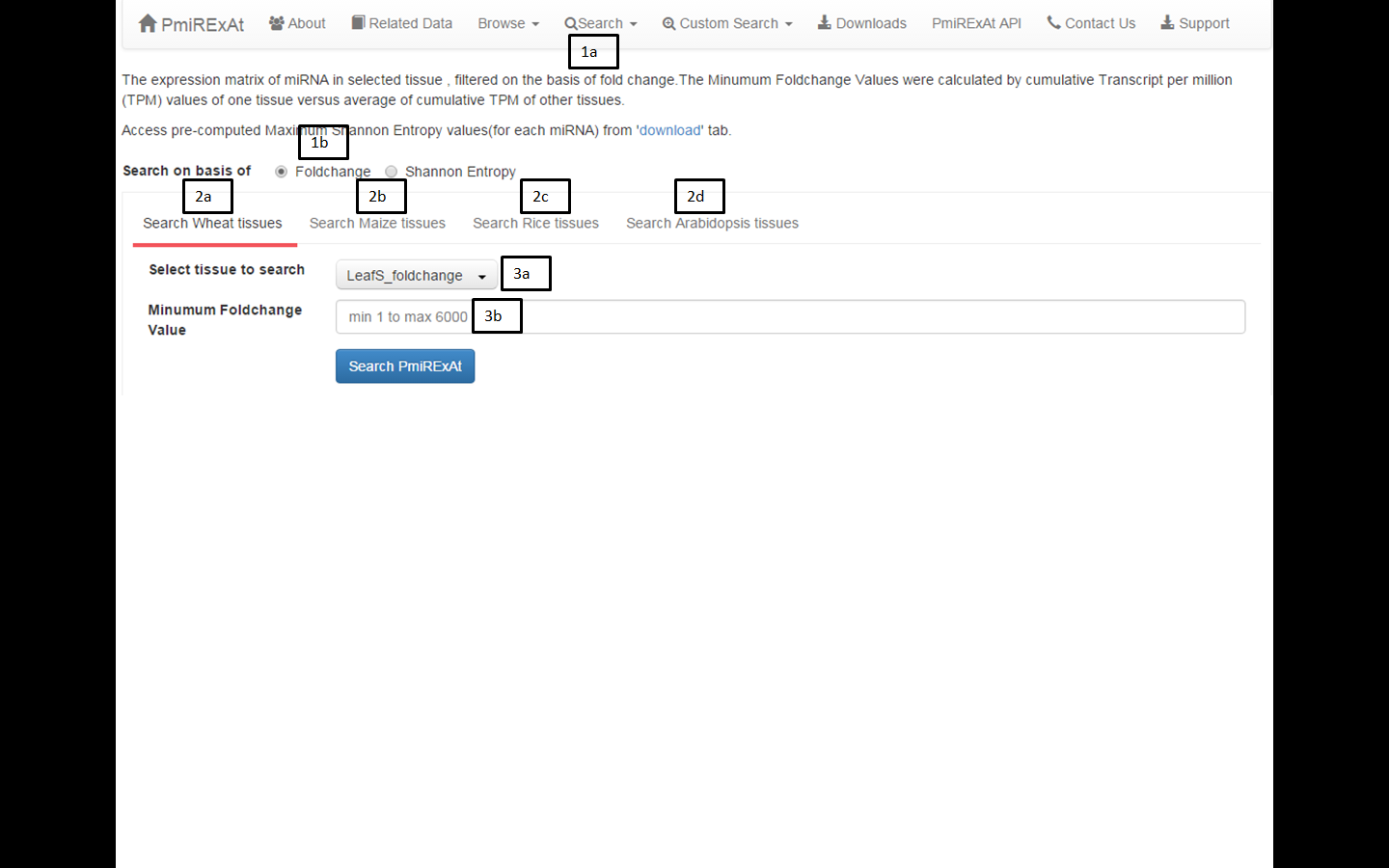


Figure 5: Screenshot showing steps to be followed for use case-5.

**Use Case-6: Filter tissue preferentially expressing miRNA on the basis of Shannon Entropy, Figure6**

**Step1-**

1. On clicking the Search button, the drop down shows options to select either I) 'Expression Search' or II) 'Tissue preferential expression in species' (1a) or III) ‘Differential Expression’. Select 'Tissue preferential expression in species' from drop down menu.
2. Select your search on the basis of Shannon Entropy (1b)

**Step2-** Click 'Search wheat tissues' (2a) or 'Search rice tissues' (2b) or 'Search maize tissues' (2c) or Search Arabidopsis tissues (2d).

**Step3-** Input Shannon Entropy value in the range of 0.1 - 3

**
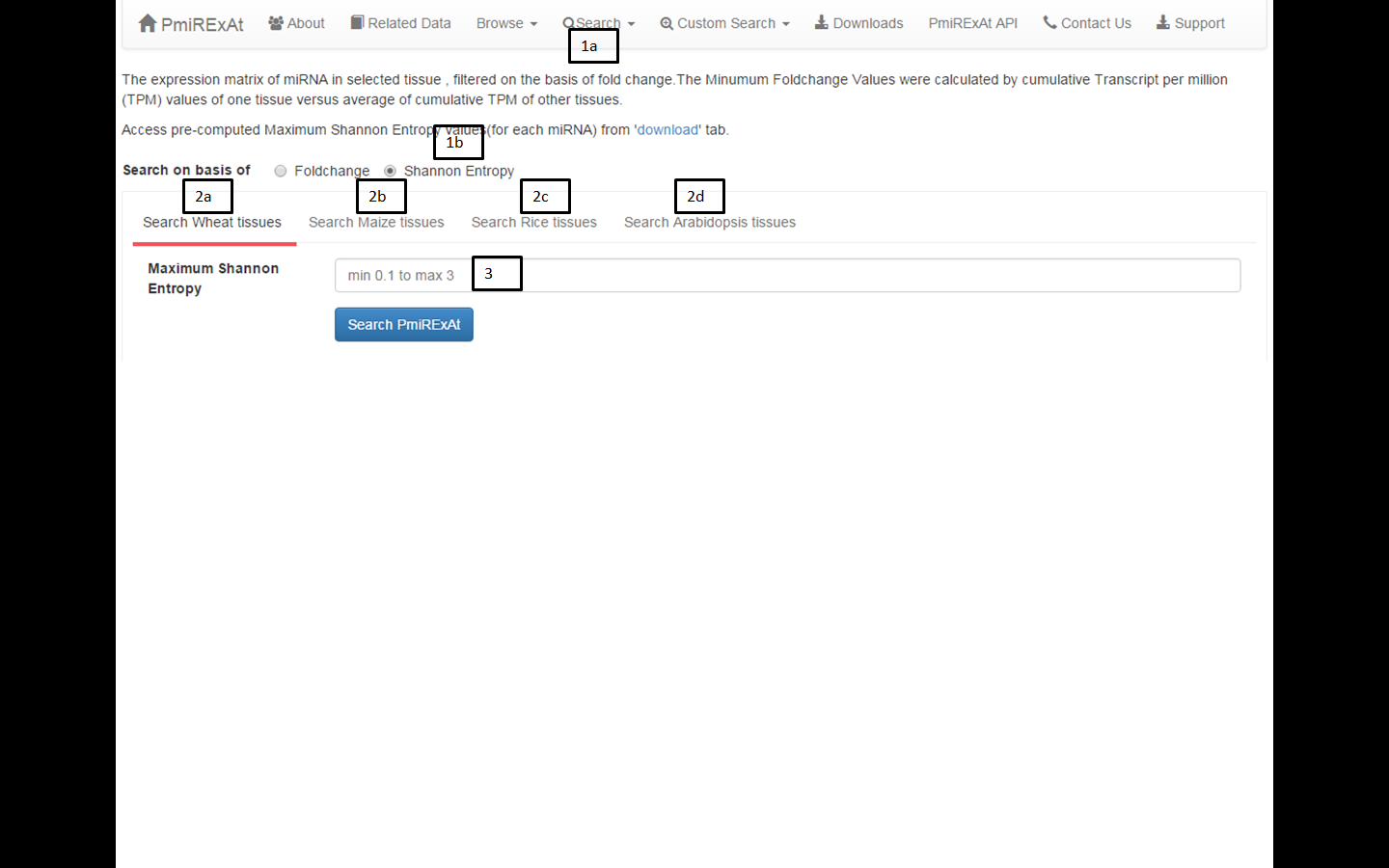
**

Figure 6: Screenshot showing steps to be followed for use case-6.

**Use Case7: Get differentially expressing miRNAs, Figure 7**

**Step1-**

1. On clicking the Search button, the drop down shows options to select either I) 'Expression Search' or II) 'Tissue preferential expression in species' or III) ‘Differential Expression’ (1). Select Differential Expression' from drop down menu.
2. Select your search on the basis of Shannon Entropy (1b)

**Step2-** Select your species of interest (2a) and the two datasets to compare (2b)

**Step3-** Results will open in lower pane (3)


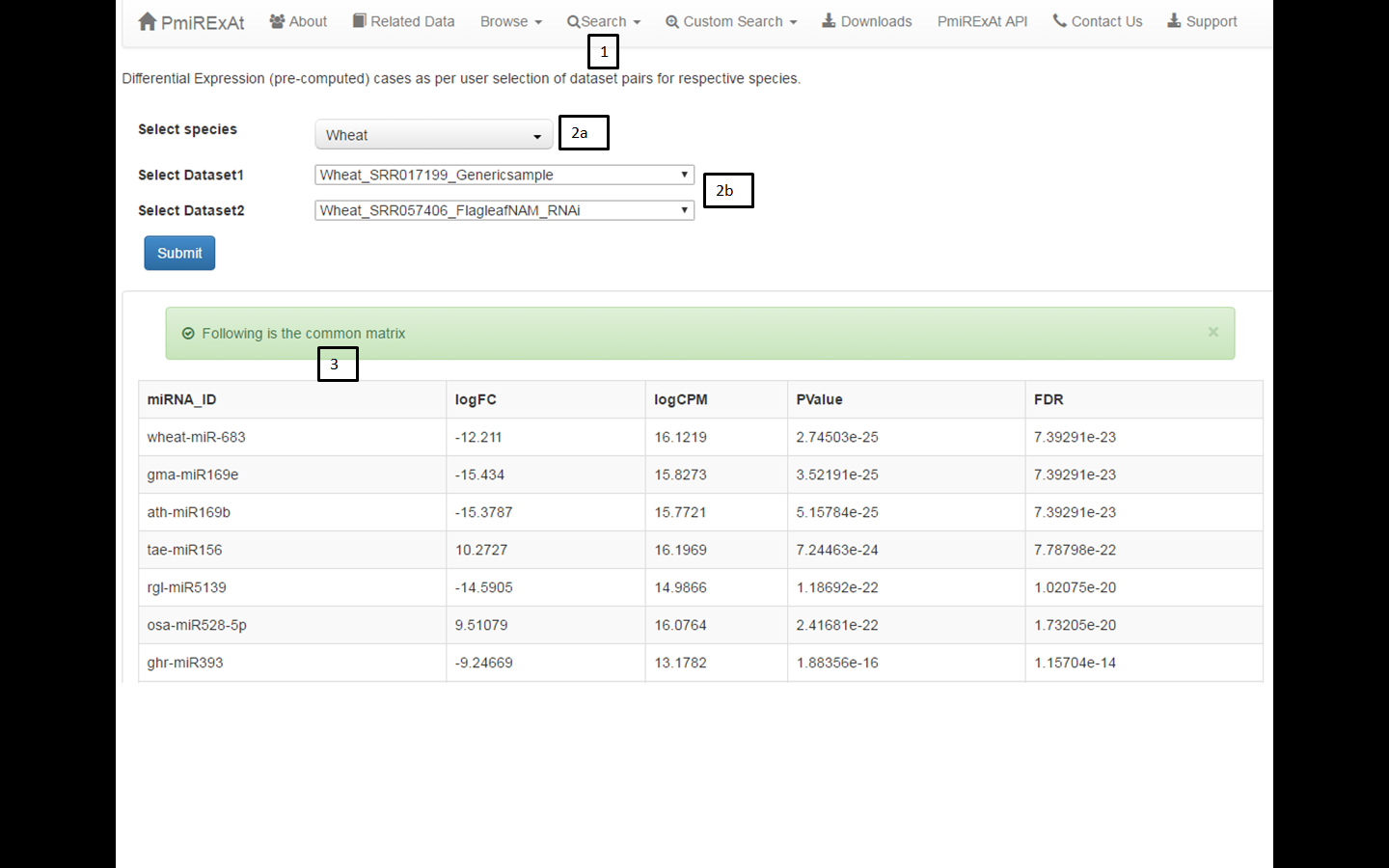


Figure7: Screenshot showing steps to follow for use case 7

**Use Case-8: Browse species wise detail of miRNA analysed and their targets in PmiRExAt, Figure8 (a, b)**

**Step1-**

1. Click 'Browse' button, the drop down shows the options, select 'By species' drop down menu (1a).
2. Click 'Wheat miRNA' (1b) or 'Rice miRNA' (1c) or 'Maize miRNA' (1d) or ‘Arabidopsis miRNA’ (1e)

**Step2-** As a result of step1 a list opens up. Here miRNA Ids are hyperlinked to their source database which will lead user to the source database of miRNA for more information about the miRNA precursor, stem loop structure, function and its target.


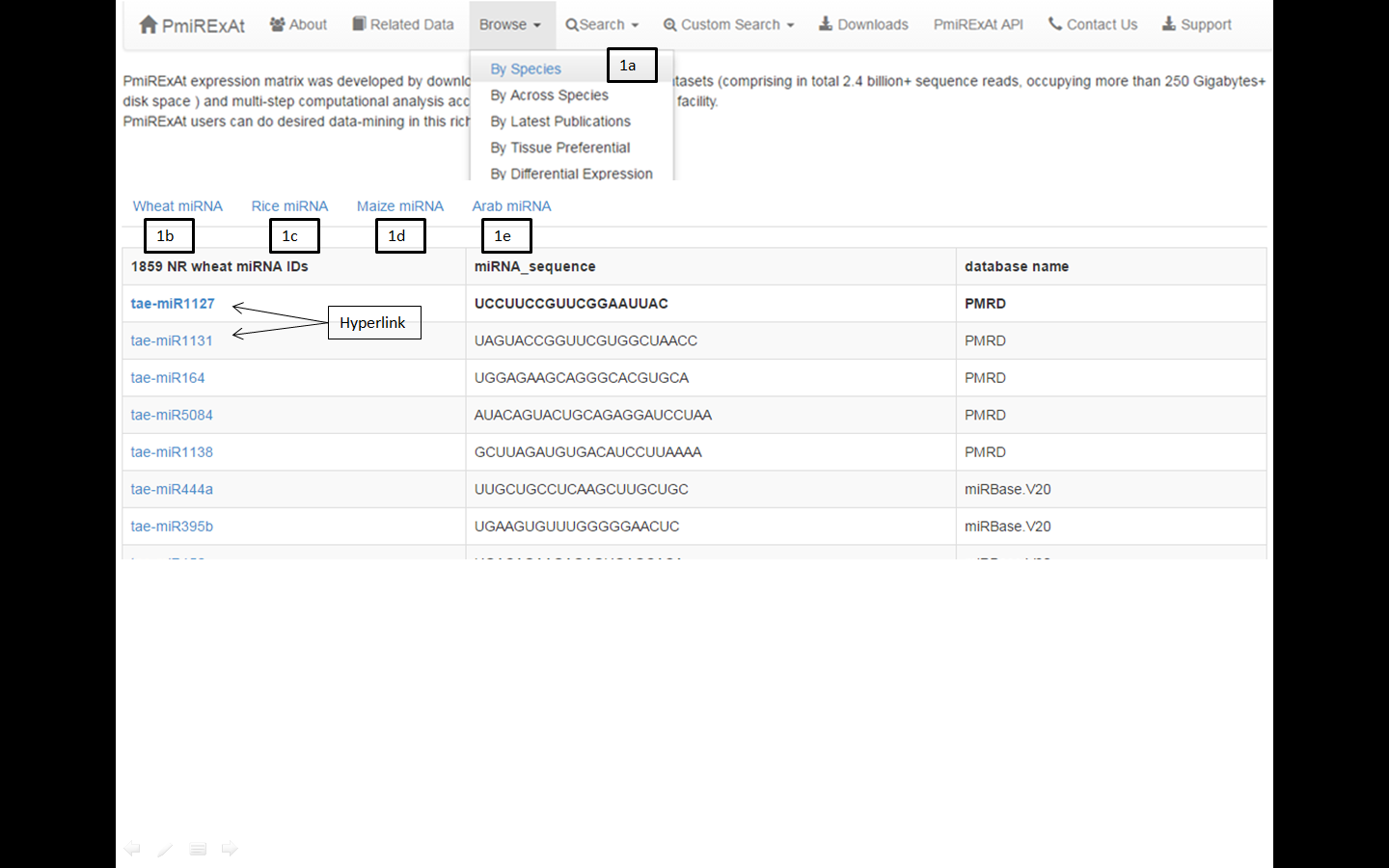


Figure8a. Screenshot showing steps to be followed for use case-8.


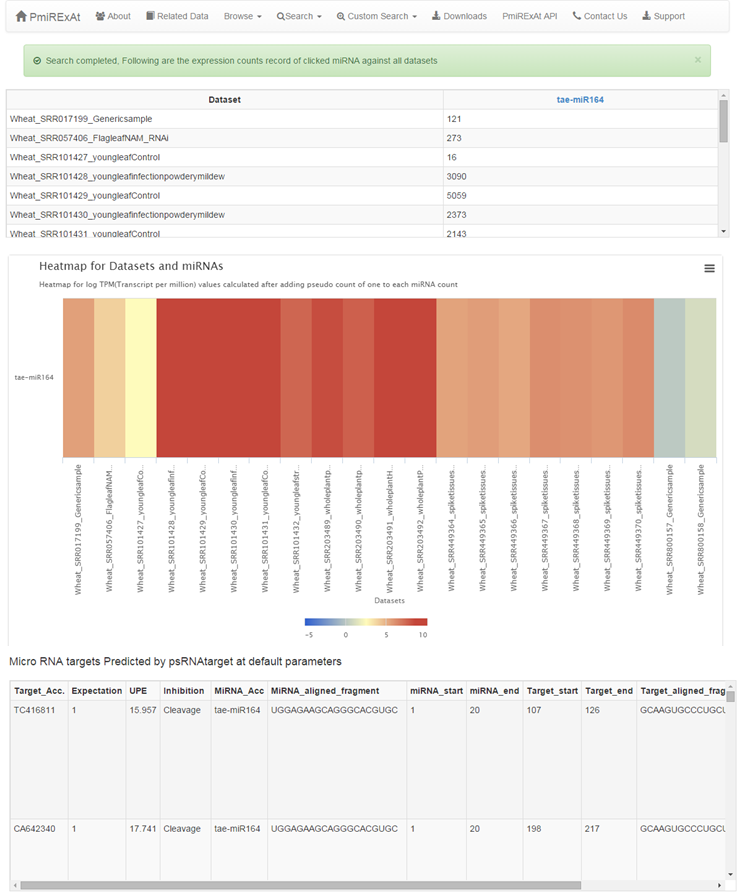


Figure8b. Screenshot showing steps to be followed for use case-8 to find targets of miRNA.

**Use Case-9: Browse across species miRNA expression matrix, Figure9**

**Step1-** Click 'Browse' button, select 'By across species' drop down menu (1).

**Step2-** Choose from the drop down option to select species of interest for comparison (2)

Now follow Step 4 to 6 as mentioned in case1.


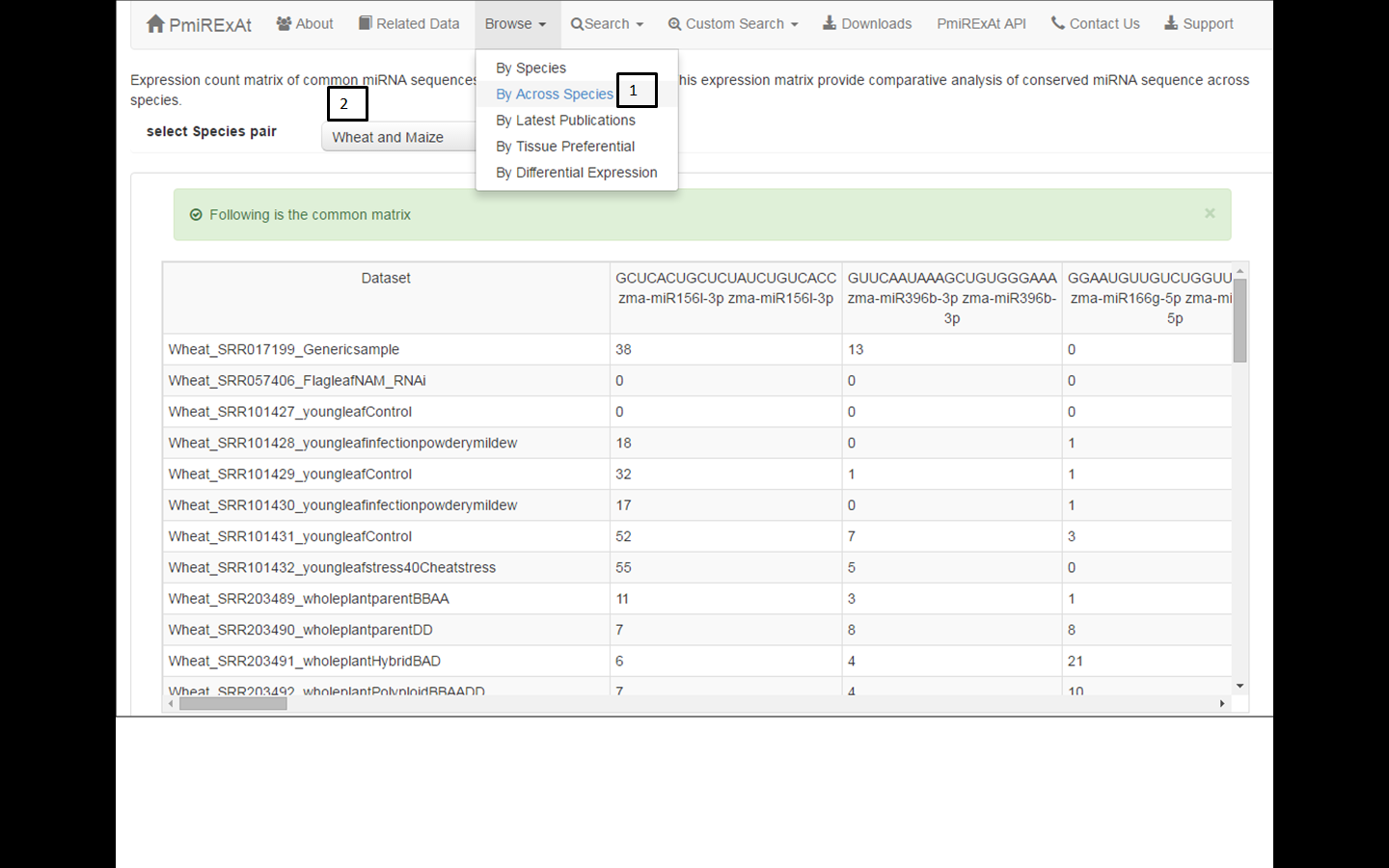


Figure 9: Screenshot showing steps to be followed for use case-9.

**Use Case 10: Browse latest publication miRNA expression, Figure10**

**Step1-** From the 'Browse' button select 'By latest publications' (1).

**Step2-** As a result of step1 a list opens up with species wise latest publications which are supported by 'show record' button (2).


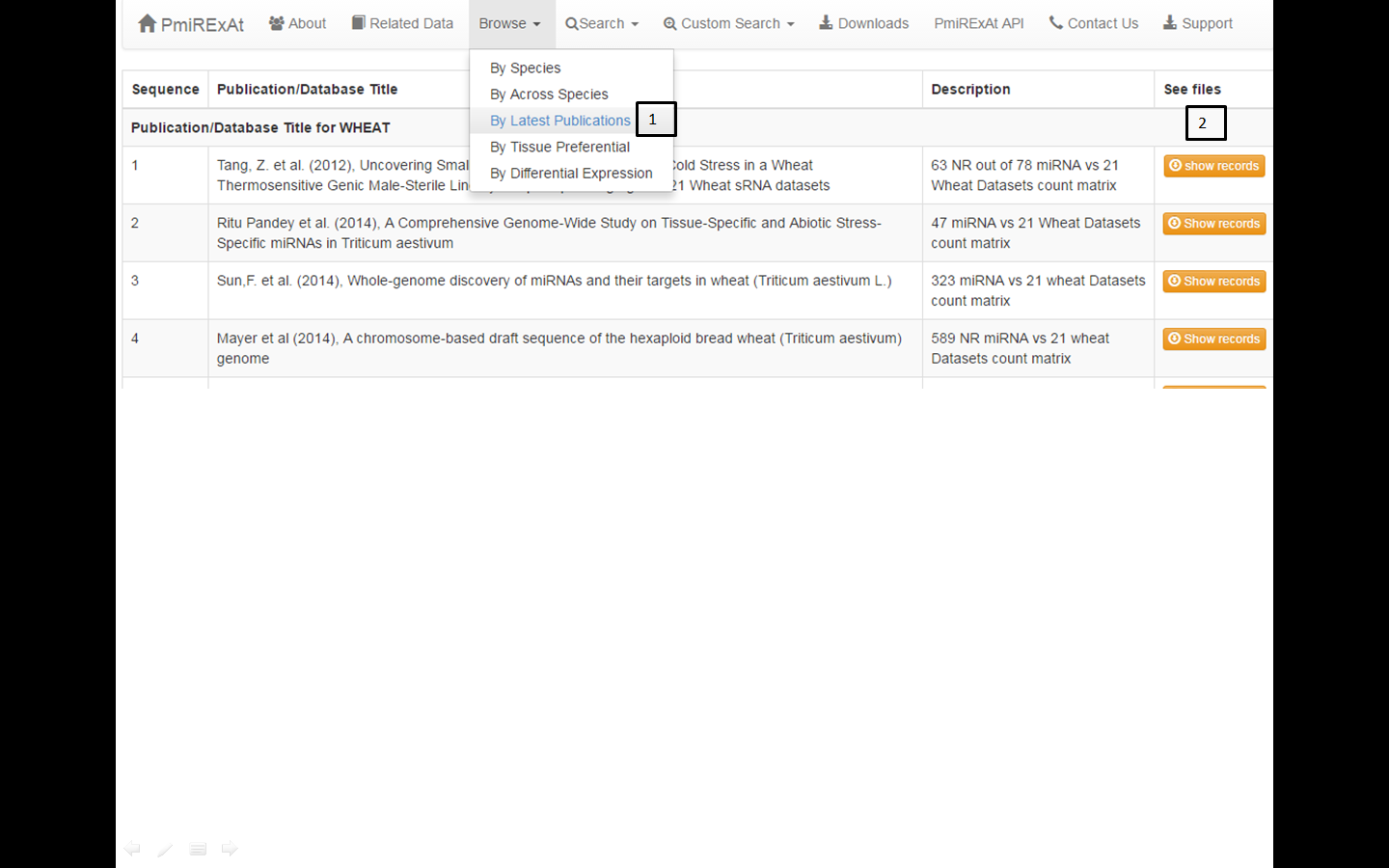


Figure 10: Screenshot showing steps to be followed for use case-10

**Use Case 11: Browse tissue preferential miRNA expression, Figure11**

**Step1-** Click 'Browse' button and select 'By Tissue Preferential' (1).

**Step2-** As a result of step1 four options of wheat (2a), maize (2b), rice (2c) and Arabidopsis (2d) are shown; select desired species and choose tissue (3) and respective miRNA to show its expression matrix and heatmap.


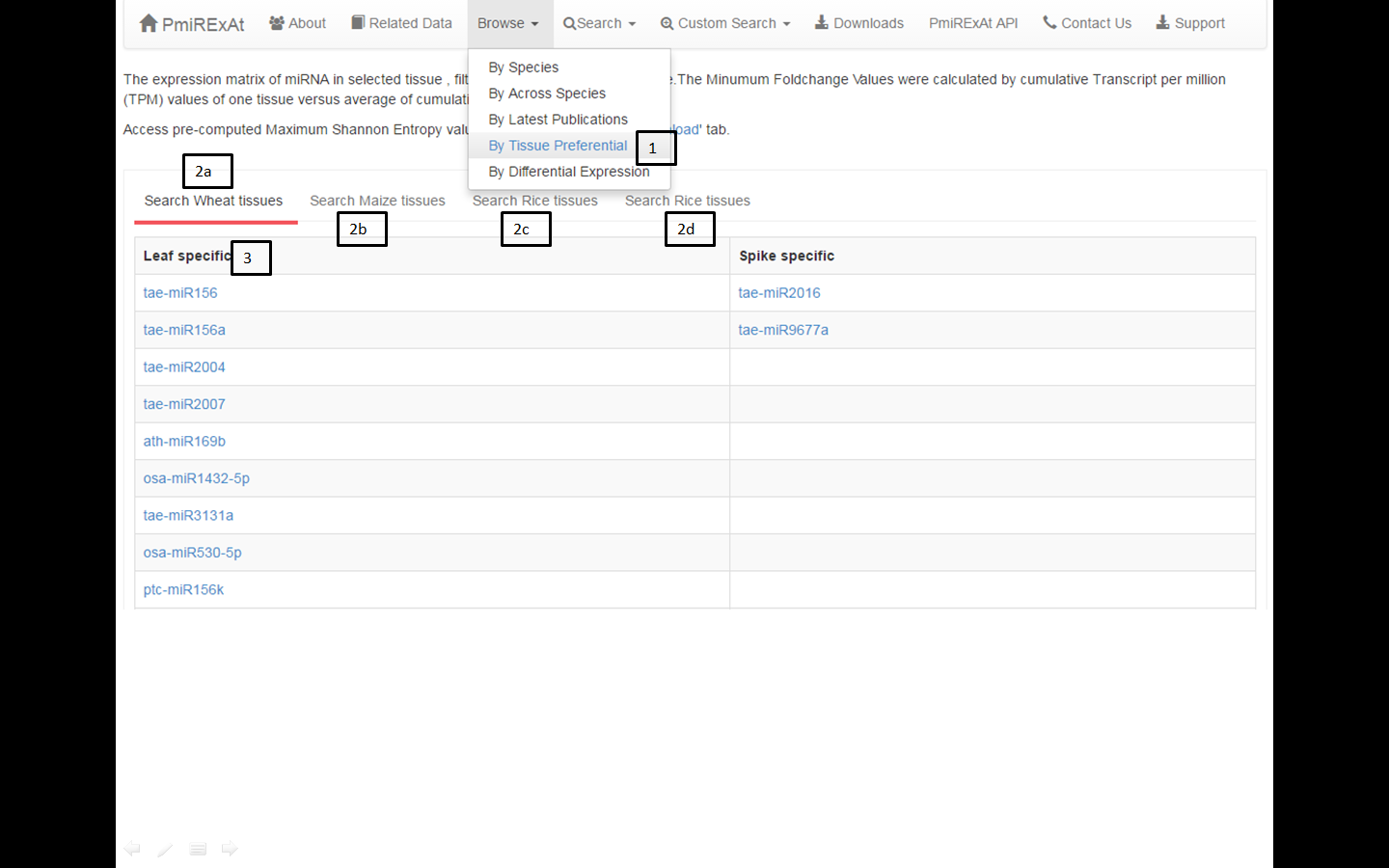


Figure 11: Screenshot showing steps to be followed for use case-11

**Use Case-12: Browse miRNA differential expression, Figure 12 (a, b)**

**Step1-** Click 'Browse' button and select 'By Differential Expression' (1).

**Step2-** Click on any link of interest (2) to open differential expression results for the same.


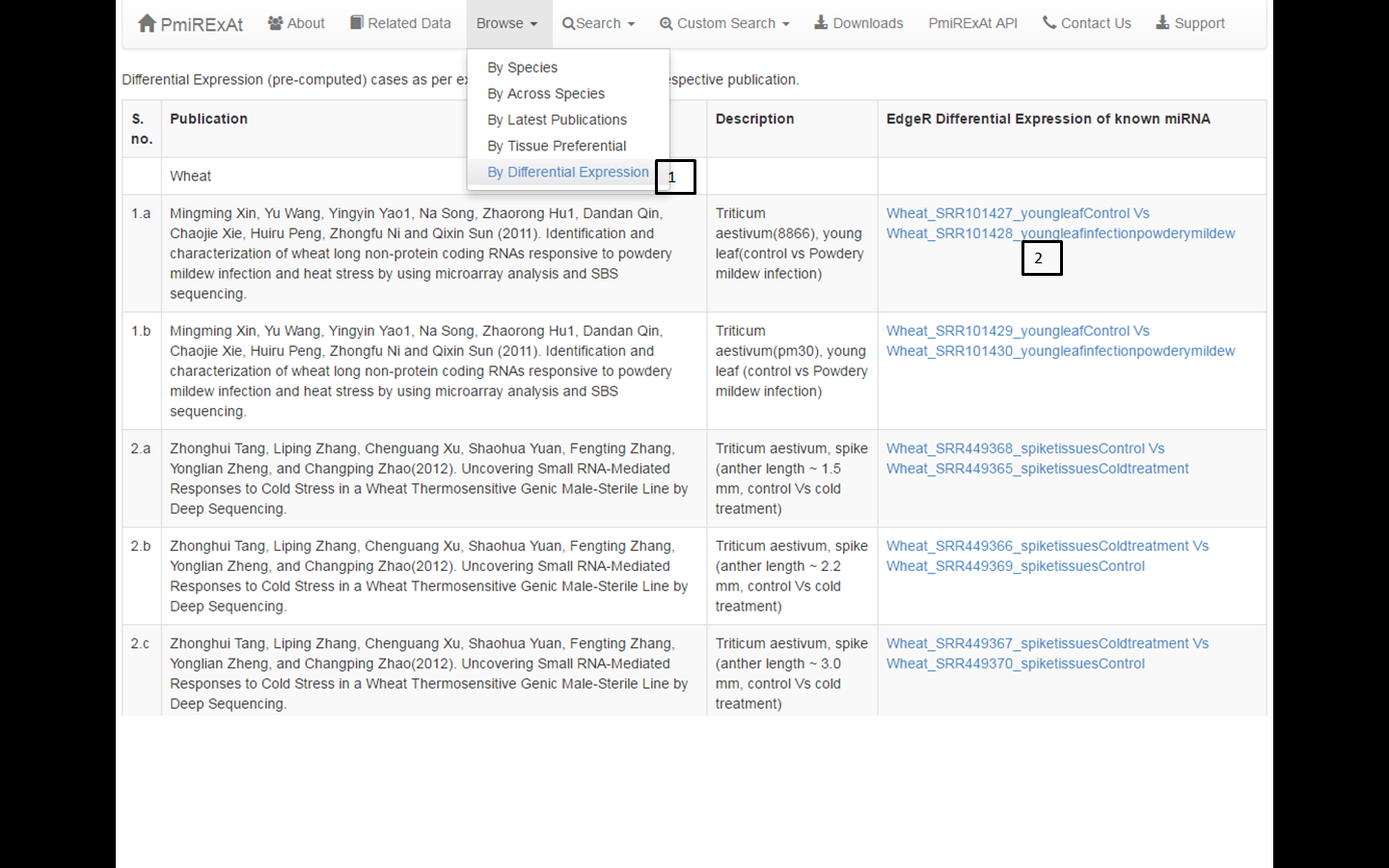


Figure 12(a): Screenshot showing steps to follow to get differential expression.


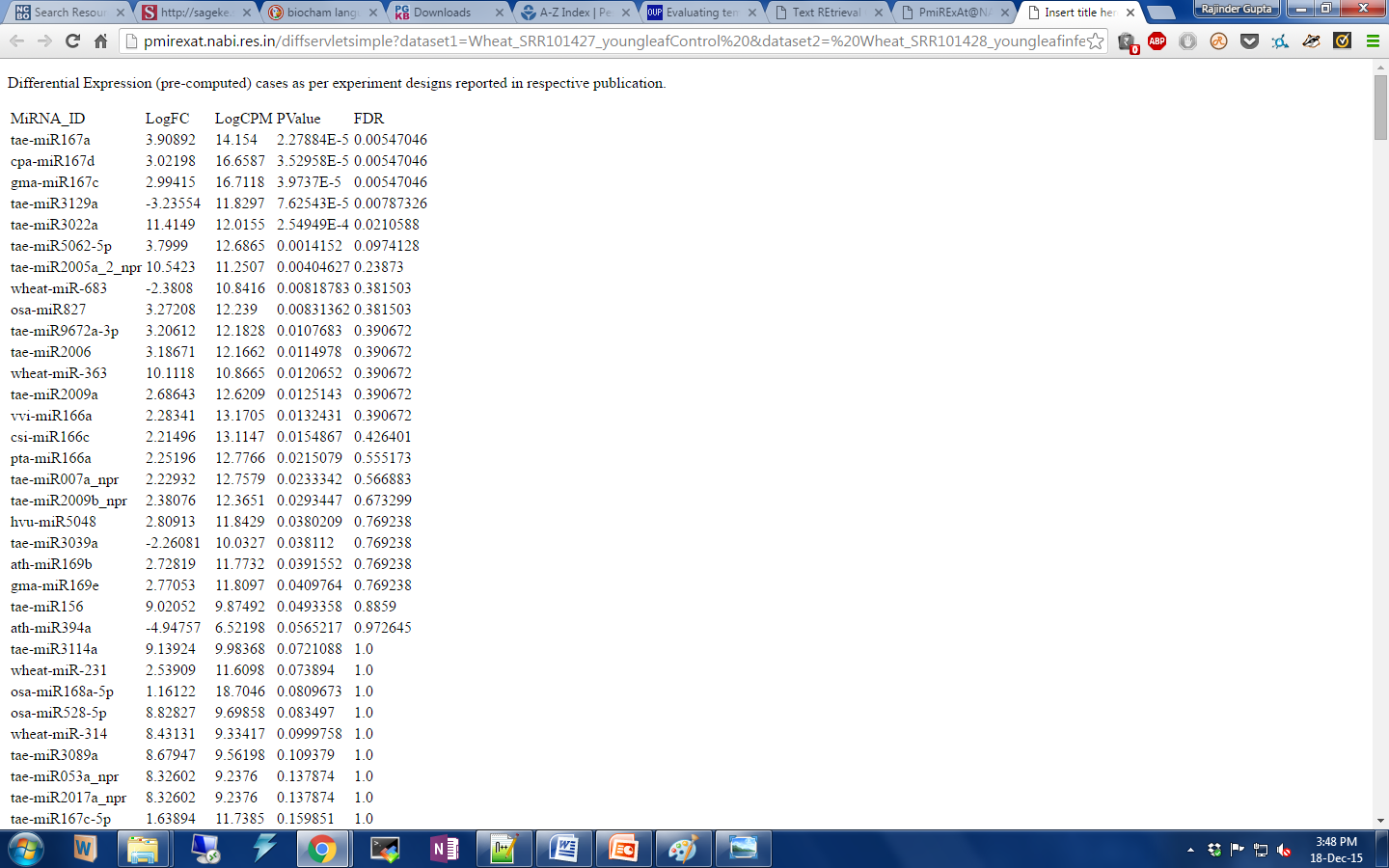


Figure 12(b): Differential expression results.

**Use Case-13: Custom search for newly detected miRNA sequence in 117 datasets, Figure13**

**Step1-** Click ''Custom search', the drop down shows the option to select either I) 'Start Search' or II) 'Search Results'. Select 'Start Search' drop down menu (1a). A form opens up, where the user can input maximum 5 sequences in fasta format by clicking 'choose file' (1b).

**Step2-** Enter a functional email ID for receiving the result (2).

**Step3-** Now user has to provide BLAST result filtration criteria's viz. percent identity (3a), mismatch (3b) and query coverage (3c) or user can choose default values (3d).

**Step4-** Click on 'Submit for search' at the end of the form.


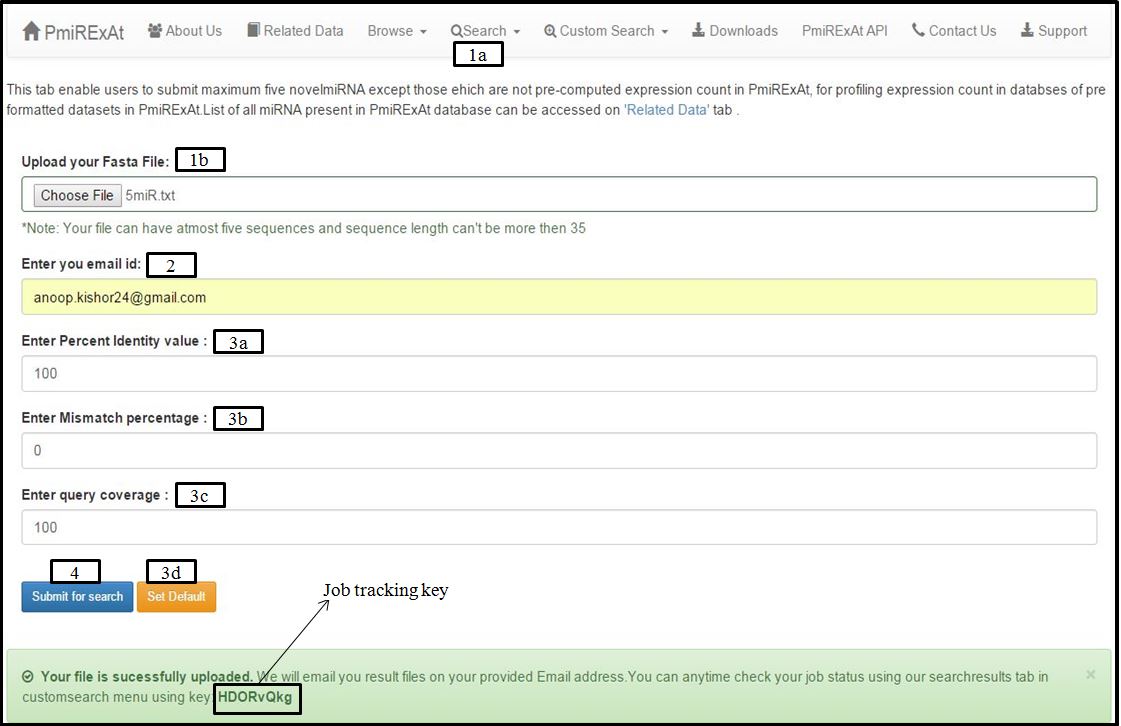


Figure 13: Screenshot showing steps to be followed for use case-13.

**Use Case 14: Custom search miRNA profiling for newly generated sRNA library, Figure14**

**Step1-** Click ''Custom search' (1a), the drop down shows the option, select 'Start Search' (1b). New user can sign up (1c) and existing one can login (1d).

**Step2-** After login, select the either 'start search' (1e) or 'check results' (1f) or 'approve sign up request' (1g).

**Step3-** Upload the SRA file in zip format (1h) and choose the desired plant species miRNA (1i) to be profiled and develop expression matrix.

**Step4-** Click on 'Submit for search' at the end of the form.


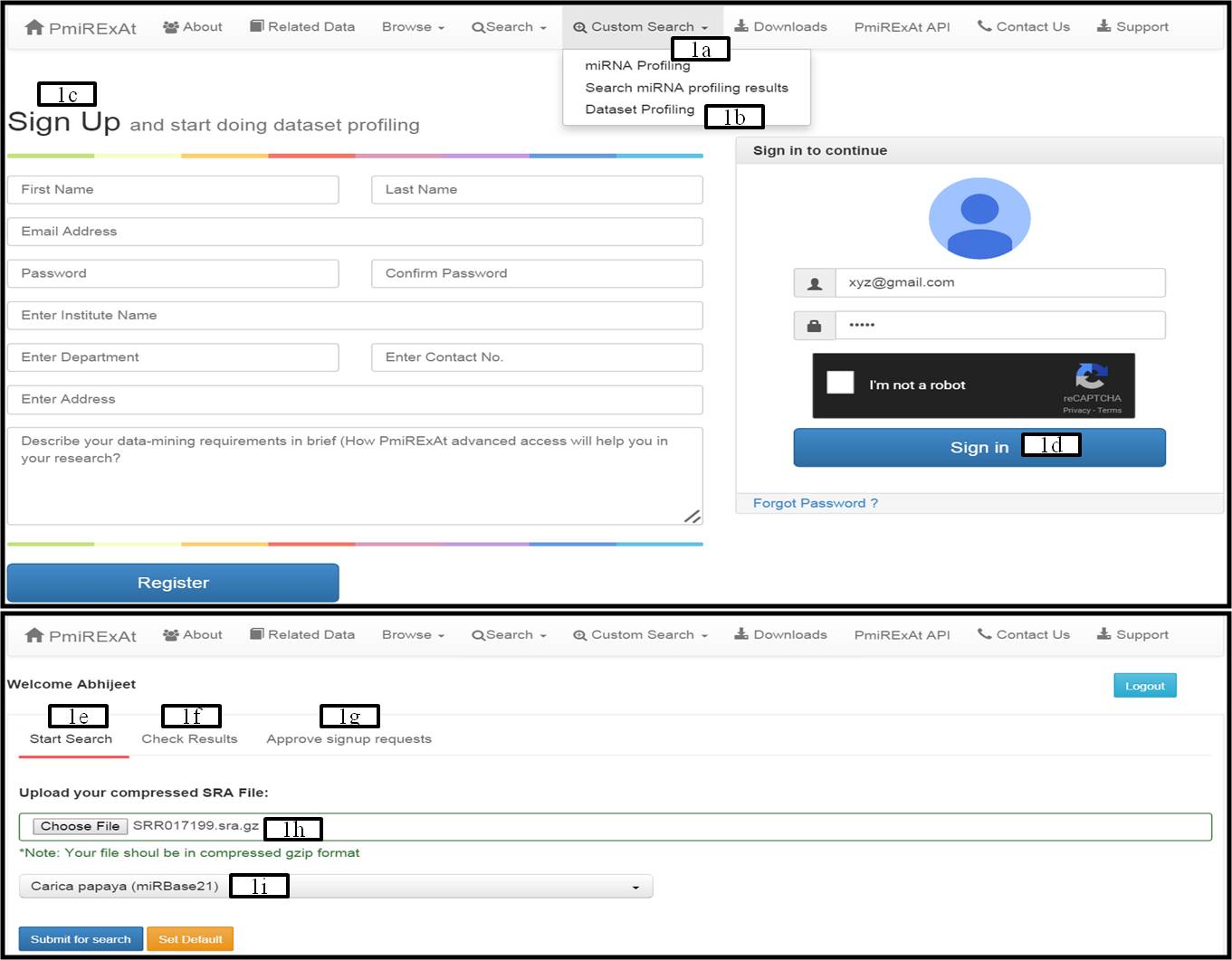


Figure 14: Screenshot showing steps to be followed for use case-11.

**Track the running job status and download the results:**

After submitting search job, interface generates a job tracking key (see figure 15), using this tracking key user can see the status of running job (figure15a) and also can download the completed job result (Figure15b).

**Step1-** Click ''Custom search', the drop down shows the option to select either I) 'Start Search' or, II) 'Search Results'. Select 'Search results' drop down menu (1).

**Step2-** Enter the key in the box (2).

**Step3a-** Click on 'Check your query status' (3). If job is still running then it will give a message that "Your file has not been processed yet. When it will be done, we will inform you by e-mail" (Figure15a).


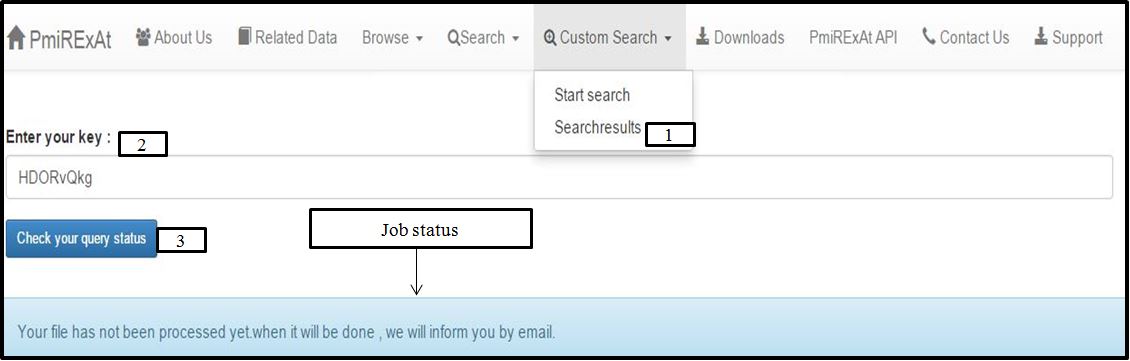


Figure 15a: Screenshot showing steps to be followed for tracking submitted job and result.

**Step3b-** Click on 'Check your query status' (3). If job is completed then it will show a count matrix and option to download the matrix (4). (Figure15b)


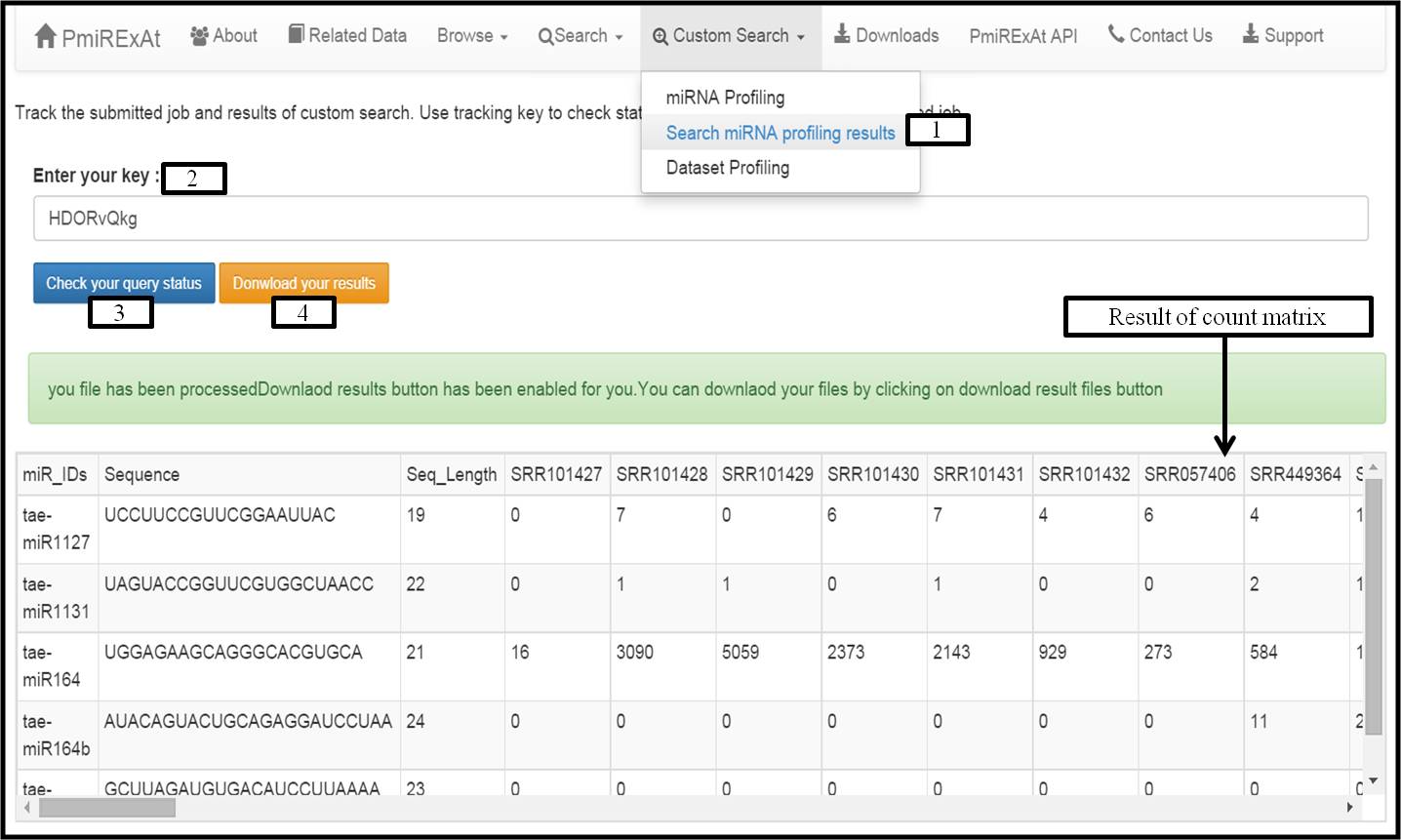


Figure 15b: Screenshot showing steps to be followed for downloading result.

**Description about tabs on interface-**

**
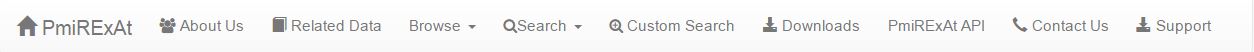
**

**PmiRExAt** : The first tab leads to the home page of interface, which gives the overview of the website.

**About us:** This tab gives the brief information of tool, development team and release information.

**Related data**: This tab provides the direct information regarding datasets like GEO accession (hyperlinked for source site of GEO for more information of datasets), SRA ids, total reads, organism, cultivar, strain, tissue, developmental stage, treatment and technique or platform and miRNA sequences analysed in this study of wheat, rice and maize.

**Browse:** This tab is enriched with the miRNA database source and sequence. Micro RNA IDs are hyperlinked for the expression in all respective species datasets with heatmap.

**Search**: User can start search by clicking on this button. As the user clicks, the interface opens up with the options to select miR ID/miR sequence, datasets/tissues and can see complete expression count matrix.

**Custom Search:** This tab enable users to submit maximum five novel miRNA except those which are not pre-computed in PmiRExAt, for profiling in databases of pre-formatted datasets and new sRNA libraries can also be profiled for PmiRExAt WRM non redundant miRs as well as other species miRs available on miRBase release 21.

**Downloads**: This tab avails all the processed data of the interface, which is used to generate the expressing tables and heatmaps.

#### PmiRExAt API: In this tab, there is link to access SOAP web service and Wsdl for api, which is helpful to other programmers/software components to connect to pmirexat api.

**Contact us**: In this tab, there are details of Principal Investigator (PI) and institutional contact.

**Support:** This tab contains help document of quick start guide for PmiRExAt and API.

1. **SOAP API and Client**

**Overview:**

PmiRExAt API is SOAP web service for PmiRExAt which allow other programmers/software components to connect to PmiRExAt. PmiRExAt API is created using Apache Cfx web services. By PmiRExAt API we have published functionalities provided by PmiRExAt web interface to other programmers who want to use already written functionalities of PmiRExAt web interface. API offers PmiRExAt application-components like getting all Sequences , Species , or to perform search of PmiRExAt database using different search criteria’s which are provided by PmiRExAt interface .

**Getting started:**

We have written Service provider or publisher for the PmiRExAt. For getting started with PmiRExAt API programmer needs to create a service consumer /Web service client for PmiRExAt API in respective platform. Wsdl for PmiRExAt API can be accessed at following URL:

[**http://pmirexat.nabi.res.in/services/ApiClassPort?wsdl**](http://pmirexat.nabi.res.in/services/ApiClassPort?wsdl)

In order to create a web client for Web Service every language/platform already has good tutorials available online. By following which any programmer can create client for respective platform/language.

Anyone who want to create web service client in java can do with Apache Cfx or Apache Axis2 using bottom to up approach .An easy tutorial for using Apache Axis 2 can be found at:

<http://www.eclipse.org/webtools/community/tutorials/BottomUpAxis2WebService/bu_tutorial.html>

PmiRExAt API provides different methods for different application components of PmiRExAt functionalities, So as soon as a web client is created for the API then by simply calling the methods one can get most of the information provided by PmiRExAt web interface.

**Methods Provided by PmiRExAt API :**

We have listed all the methods with brief description, parameters required by method and return type for the method provided by API on PmiRExAt API tab :

| **Method Prototype** | **Description** | **Parameters** | **Return Type** |
| --- | --- | --- | --- |
| getAllWheatMirs() | Return all wheat miRs available at PmiRExAt database | No parameters | List |
| getAllMaizeMirs() | Return all maize miRs available at PmiRExAt database | No parameters | List |
| getAllRiceMirs() | Return all rice miRs available at PmiRExAt database | No parameters | List |
| getAllWheatSequence() | Returns all Wheat sequence available at PmiRExAt database | No parameters | List |
| getAllMaizeSequence() | Returns all Maize sequence available at PmiRExAt database | No parameters | List |
| getAllRiceSequence() | Returns all Rice sequence available at PmiRExAt database | No parameters | List |
| getAllRiceDatasets() | To get all rice dataset available at PmiRExAt database | No parameters | List |
| getAllWheatDatasets() | To get all wheat dataset available at PmiRExAt database | No parameters | List |
| getAllMaizeDatasets() | To get all maize dataset available at PmiRExAt database | No parameters | List |
| getmiRidbysequene(String sequence) | To get miRNA ids for a sequence available in PmiRExAt database | String sequence (Takes sequence as parameter) Sequence should exist in PmiRExAt dataset records | List |
| findmiRidbysequenceinWheat(String sequence) | To get miRNA id for a sequence available in PmiRExAt wheat database | String Sequence(Takes sequence as parameter) : sequence should exist in PmiRExAt Wheat dataset records | String |
| findmiRidbysequenceinMaize(String sequence) | To get miRNA id for a sequence available in PmiRExAt maize database | String Sequence(Takes sequence as parameter): sequence should exist in PmiRExAt maize dataset records | String |
| findmiRidbysequenceinRice(String sequence) | To get miRNA id for a sequence available in PmiRExAt rice database | String Sequence(Takes sequence as parameter) : sequence should exist in PmiRExAt rice dataset records | String |
| getSequencebymiRid(String miRid) | To find sequence for a miRNA id available in PmiRExAt database | String miRNA id (Takes miRNA id as parameter) : miRNA id should exist in PmiRExAt dataset records | String |
| searchdsbymiRid(String miRid,String Dataset) | To get expression count for datasets by miRNA id | String miRid (miRNA id),String Dataset(dataset name ) : miRNA id should exist in PmiRExAt records | Integer |
| searchWheatDbbySequence(String sequence,String dataset) | To get expression count in wheat dataset by sequence | String sequence(sequence),String dataset(dataset name) : sequence and dataset name should exist in the wheat dataset | Integer |
| searchRiceDbbySequence(String sequence,String dataset) | To get expression count in rice dataset by sequence | String sequence(sequence),String dataset(dataset name) : sequence and dataset name should exist in the wheat dataset | Integer |
| searchMaizeDbbySequence(String sequence,String dataset) | To get expression count in maize dataset by sequence | String sequence(sequence),String dataset(dataset name) :sequence and dataset name should exist in the wheat dataset | Integer |
| getalltisssuesinwheat() | To get list of all tissues present in wheat datasets | No parameters | List |
| getalltissuesinMaize() | To get list of all tissues present in maize datasets | No parameters | List |
| getalltissuesinRice() | To get list of all tissues present in rice datasets | No parameters | List |
| getdsfortissueinwheat(String tissue) | To get list of all datasets of a specific tissue in wheat | String tissue(tissue name ) : should exist in wheat dataset | List |
| getdsfortissueinMaize(String tissue) | To get list of all datasets of a specific tissue in maize | String tissue(tissue name ): should exist in maize dataset | List |
| getdsfortissueinRice(String tissue) | To get list of all datasets of a specific tissue in rice | String tissue(tissue name) :should exist in rice dataset | List |

Please see ‘PmiRExAt Methods’ heading on: <http://pmirexat.nabi.res.in/api.html> page.

**Sample Code:**

This section has sample code for using PmiRExAt API in Java as we have written our web service test client in Java but anyone can read this and can get an idea about using the API. As on any other platform method calls will be same but with different syntax.

First we need to create object for the proxy object provided by web client.

//ApiClassService (automatically created proxy object created in webclient , Name of the class will be different)

ApiClassService wc_obj = new ApiClassService();

//To get portobject by which all operation of webservice can be called

PmiRExAtSEI operation = wc_obj.getApiClassPort();

As we have received reference for port object we can call the methods using this object.

// To receive all wheat sequences present in PmiRExAt API

List<String> Wheatsequences = operation.getAllWheatSequence();

//To get miRNA id for a sequence

List<String> searchresult = operation.getmiRidbysequene("UAGUACCGGUUCGUGGCUAACC");

//To get all datasets present for rice in PmiRExAt Database

List<String> Ricedatasets = operation.getAllRiceDatasets();

//To get expression count for a miRNA id for a dataset (programmer should ensure that using other methods that whether to be searched miRNA id and given dataset exist for same species by using other methods which provide list of all datasets and miRNA ids in a particular species)

int expression_count = operation.searchdsbymiRid("tae-miR159a","Wheat_SRR057406_FlagleafNAM_RNAi");

//As PmiRExAt provides functionality to do tissue based search, So one can get tissues in a species and can get datasets in it.

//to get list of tissues in Wheat species

List<String> wheattissues = operation.getalltisssuesinwheat();

//To get list of datasets in a species, following will return all datasets in leaf tissue

List<String> leaf_datasets = operation.getdsfortissueinwheat("leaf");

We have already written a sample java web service client which is available to download on PmiRExAt interface at bottom. For reference you can download the zip file and can set up it on your IDE . We have tried to keep it simple and it has comments available with the code.

1. **Contact us**

Feel free to contact us with questions, bugs, suggestions, or anything else related to PmiRExAt. Email: ict@nabi.res.in, shrikant@nabi.res.in.

**Development team:**

Anoop Kishor Singh Gurjar (anoop.kishor24@gmail.com, anoop@nabi.res.in)

Abhijeet Singh Panwar (abhijeetpnwr@gmail.com)

Rajinder Gupta (rajinder@nabi.res.in)

Shrikant S Mantri (shrikant@nabi.res.in, bioinfoman3@gmail.com)

**Affiliation:**

**1)** National Agri Food Biotechnology Institute (NABI), Mohali, Punjab, INDIA.

**2)** Center for Development of Advance Computing (C-DAC), Pune, Maharashtra, INDIA.

**Principal Investigator:**

Shrikant S Mantri,

Scientist C (Computational Biology),

National Agri-Food Biotechnology Institute (NABI)

(Department of Biotechnology, Government of India)

C-127, Industrial Area, Phase VIII, S.A.S. Nagar, Mohali

Punjab, India-160071

TEL {Office}:+91(172)4990104

Fax: +91 172 4604888

Email: shrikant@nabi.res.in, bioinfoman3@gmail.com

URL: www.nabi.res.in

1. **References**

Camacho, C. et al. (2009) BLAST+: architecture and applications. BMC Bioinformatics, 10, 421.

Kadota, K. et al. (2006) ROKU: a novel method for identification of tissue-specific genes. BMC Bioinformatics, **7**, 294.

Tarazona, S. *et al.* (2011) Differential expression in RNA-seq: A matter of depth. *Genome Res.*, **21**, 2213–2223.

Guo, Z. *et al.* (2014) Genome-wide survey of tissue-specific microRNA and transcription factor regulatory networks in 12 tissues. *Sci. Rep.*, **4**, 5150.

**Websites:**

http://www.ncbi.nlm.nih.gov/SRA.

http://hannonlab.cshl.edu/fastx_toolkit/index.html by Hannon Lab

[http://www.highcharts.com](http://www.highcharts.com/)

<http://getbootstrap.com/>

<https://developers.google.com/maps/>

<http://fortawesome.github.io/Font-Awesome/>

<https://github.com/aehlke/tag-it>

<http://jqueryui.com/>

<http://www.bootstrap-switch.org/>

http://www.broadinstitute.org/cancer/software/morpheus/
