# Supplementary material for: PmiRExAt: plant miRNA expression atlas database and web applications
Source: Database (Oxford). 2016 Apr 13;2016:baw060. doi: 10.1093/database/baw060 (PMC4830907; doi:10.1093/database/baw060)
Supplement: Supplementary Data [file supp_baw060_suppl_data.zip › supplementary_captions.docx]

**List of Supplementary Files**

Supplementary files:

1. Figure S1: miRNA size distribution. MicroRNA length distributed on x-axis between 17-28 nt and expression frequency on y-axis. a) Wheat 21 libraries miRNA size distribution, b) Rice 53 libraries miRNA size distribution and, c) Maize 43 libraries miRNA size distribution,
2. Figure S2: Expression heatmaps (arranged according to ordinal classes), each library normalized expression data (TPM) was sorted on ordinal basis and distributed in 10 categories according to the respective species miRNA numbers. The heatmaps were developed for each species (a: wheat, b: rice, c: maize) showing category 1st to 10th.
3. Figure S3: Abundance of conserved miRNA. Normalized expression values (i.e. TPM) were cumulated for each species and represented in bar graph format to show expression of conserved miRNA of WRM.
4. Figure S4: Composition and length distribution of conserved miRNAs between W, R and M The x-axis represents length (nt) of miRNA and frequency of miRNAs on y-axis.

Supplementary tables:

1. Supplementary Table S1: Dataset and miR details.

Sheet 1: Information about wheat 21 datasets,

Sheet 2: Information about rice 53 datasets,

Sheet3: Information about maize 43 datasets,

Sheet 4: Information about 1859 NR miR sequences of wheat,

Sheet 5: Information about 2330 NR miR sequences of rice and,

Sheet 6: Information about 283 NR miR sequences of maize,

Sheet 7: List of 51 conserved miRNA between WRM.

1. Supplementary Table S2: Expression level category based on TPM values. Each dataset expression values of normalized data were sorted according to ordinal basis.

Sheet 1: Expression matrix of ordinal sorted wheat miRs in 21 datasets (TPM values),

Sheet 2: Matrix of ordinal sorted wheat miRs in 21 datasets (miRNA Ids),

Sheet3: Expression Matrix of ordinal sorted rice miRs in 53 datasets (TPM values),

Sheet 4: Matrix of ordinal sorted rice miRs in 53 datasets (miRNA Ids),

Sheet 5: Expression Matrix of ordinal sorted maize miRs in 43 datasets (TPM values),

Sheet 6: Matrix of ordinal sorted maize miRs in 43 datasets (miRNA Ids).

1. Supplementary Table S3: psRNATarget predicted miRNA targets for WRM.

Sheet 1: Information about wheat 1859 miRNA targets,

Sheet 2: Information about rice 2330 miRNA targets,

Sheet3: Information about maize 283 miRNA targets.

1. Supplementary Table S4: Conserved miRNA correlation (Pearson correlation) matrix between all 117 datasets of WRM.
2. Supplementary Table S5: miRNA abundance matrixes, Shannon entropy and fold change values and edgeR calculated values.

Sheet 1: Wheat 1859 miRNA versus 21 wheat datasets expression count matrix with fold change, edgeR calculated values and Shannon entropy column,s

Sheet 2: Rice 2330 miRNA versus 53 rice datasets expression count matrix with fold change, edgeR calculated values and Shannon entropy column and,

Sheet 3: Maize 283 miRNA versus 43 maize datasets expression count matrix with fold change, edgeR calculated values and Shannon entropy column.

Supplementary doc file 1: Quick start guide. This file explains the use cases of PmiRExAt.
