# Supplementary figures and images for: PmiRExAt: plant miRNA expression atlas database and web applications
Source: Database (Oxford). 2016 Apr 13;2016:baw060. doi: 10.1093/database/baw060 (PMC4830907; doi:10.1093/database/baw060)

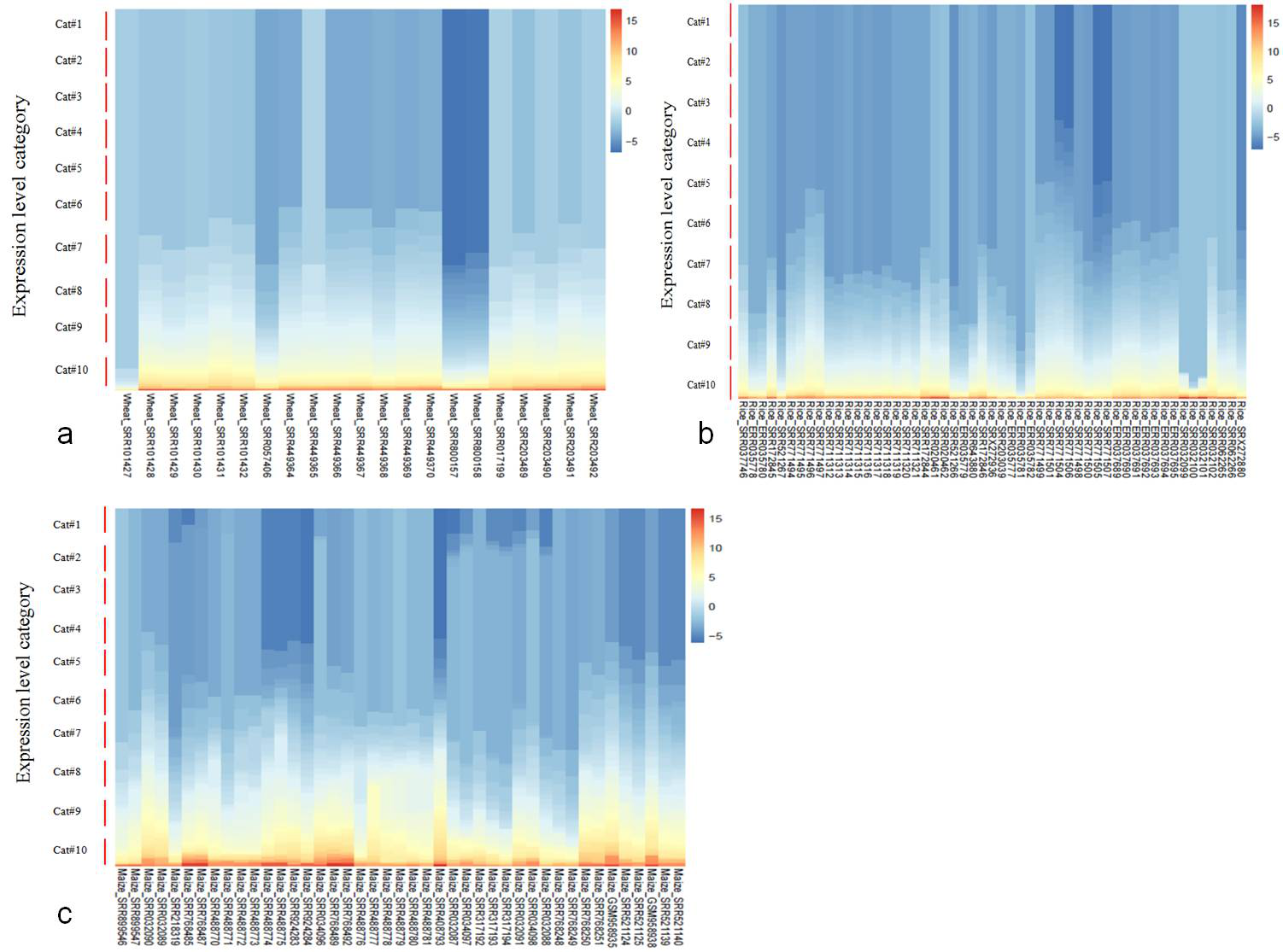

Supplement: Supplementary Data [file supp_baw060_suppl_data.zip › Figure S2.tif]

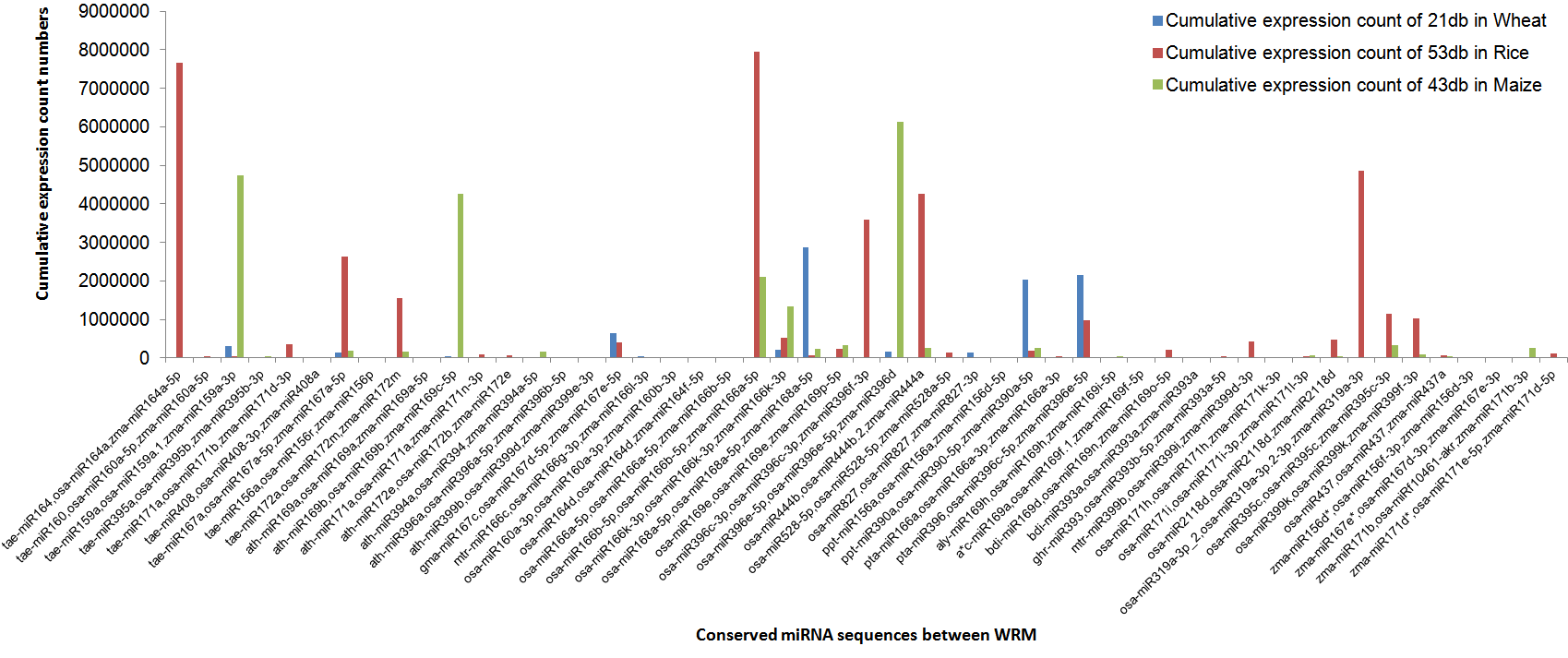

Supplement: Supplementary Data [file supp_baw060_suppl_data.zip › Figure S3.tif]

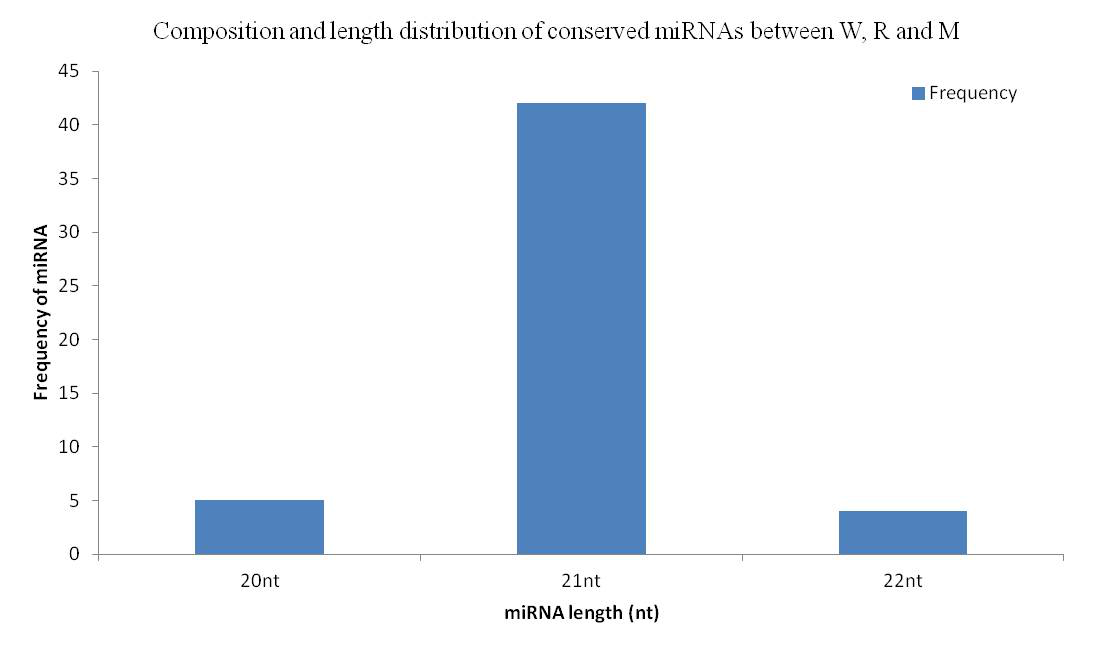

Supplement: Supplementary Data [file supp_baw060_suppl_data.zip › Figure S4.tif]

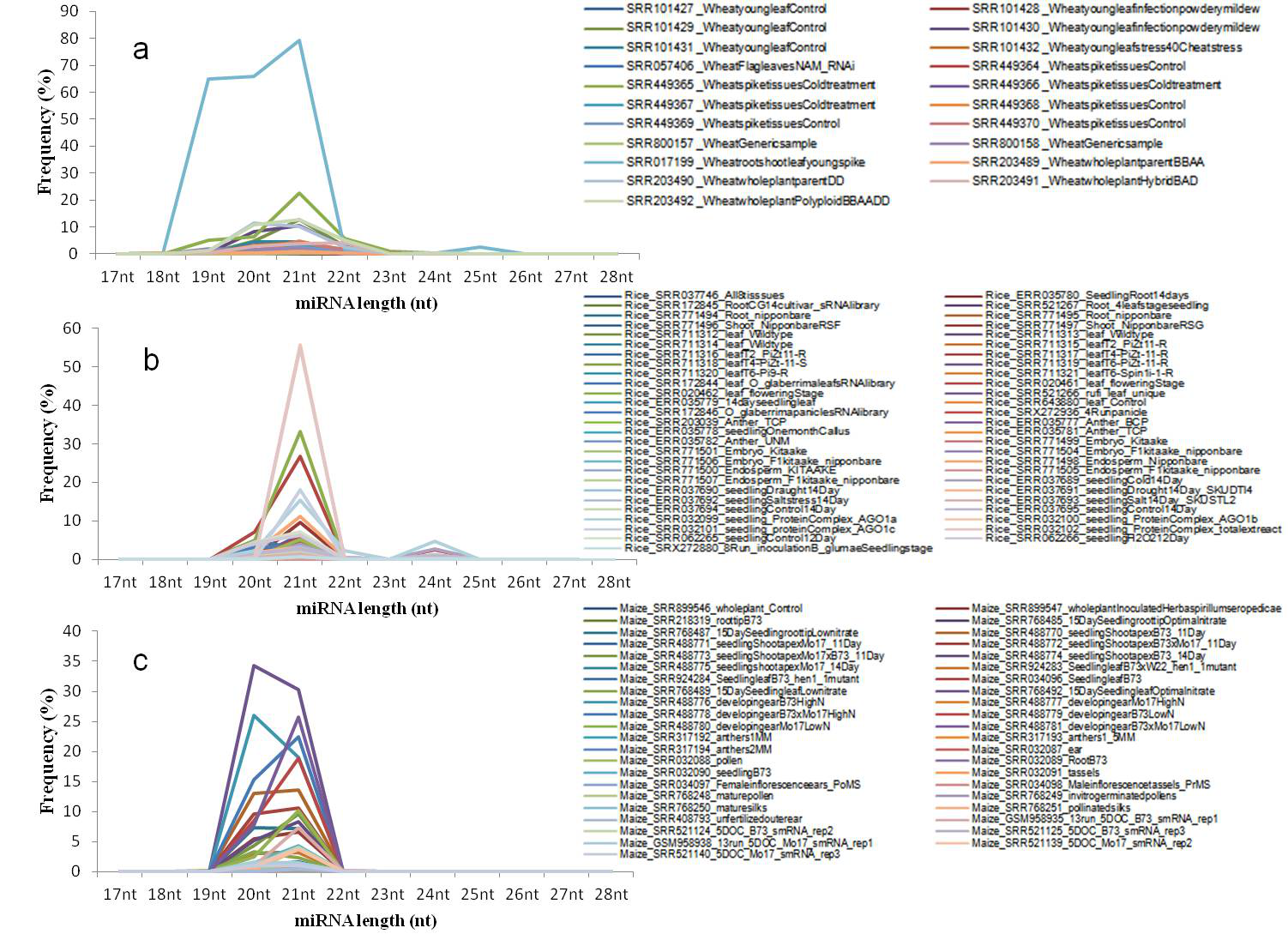

Supplement: Supplementary Data [file supp_baw060_suppl_data.zip › Figure S1.tif]
